# Electron Tomography of HIV-1 Infection in Gut-Associated Lymphoid Tissue

**DOI:** 10.1371/journal.ppat.1003899

**Published:** 2014-01-30

**Authors:** Mark S. Ladinsky, Collin Kieffer, Gregory Olson, Maud Deruaz, Vladimir Vrbanac, Andrew M. Tager, Douglas S. Kwon, Pamela J. Bjorkman

**Affiliations:** 1 Division of Biology & Biological Engineering 114-96, California Institute of Technology, Pasadena, California, United States of America; 2 Ragon Institute of MGH, MIT and Harvard, Cambridge, Massachusetts, United States of America; 3 Center for Immunology and Inflammatory Diseases, Massachusetts General Hospital and Harvard Medical School, Charlestown, Massachusetts, United States of America; 4 Howard Hughes Medical Institute, California Institute of Technology, Pasadena, California, United States of America; Institut Pasteur, France

## Abstract

Critical aspects of HIV-1 infection occur in mucosal tissues, particularly in the gut, which contains large numbers of HIV-1 target cells that are depleted early in infection. We used electron tomography (ET) to image HIV-1 in gut-associated lymphoid tissue (GALT) of HIV-1–infected humanized mice, the first three-dimensional ultrastructural examination of HIV-1 infection in vivo. Human immune cells were successfully engrafted in the mice, and following infection with HIV-1, human T cells were reduced in GALT. Virions were found by ET at all stages of egress, including budding immature virions and free mature and immature viruses. Immuno-electron microscopy verified the virions were HIV-1 and showed CD4 sequestration in the endoplasmic reticulum of infected cells. Observation of HIV-1 in infected GALT tissue revealed that most HIV-1–infected cells, identified by immunolabeling and/or the presence of budding virions, were localized to intestinal crypts with pools of free virions concentrated in spaces between cells. Fewer infected cells were found in mucosal regions and the lamina propria. The preservation quality of reconstructed tissue volumes allowed details of budding virions, including structures interpreted as host-encoded scission machinery, to be resolved. Although HIV-1 virions released from infected cultured cells have been described as exclusively mature, we found pools of both immature and mature free virions within infected tissue. The pools could be classified as containing either mostly mature or mostly immature particles, and analyses of their proximities to the cell of origin supported a model of semi-synchronous waves of virion release. In addition to HIV-1 transmission by pools of free virus, we found evidence of transmission via virological synapses. Three-dimensional EM imaging of an active infection within tissue revealed important differences between cultured cell and tissue infection models and furthered the ultrastructural understanding of HIV-1 transmission within lymphoid tissue.

## Author Summary

HIV/AIDS remains a global public health problem with over 33 million people infected worldwide. High-resolution imaging of infected tissues by three-dimensional electron microscopy can reveal details of the structure of HIV-1, the virus that causes AIDS, how it infects cells, and how and where the virus accumulates within different tissue sub-structures. Three-dimensional electron microscopy had previously only been performed to image infected cultured cells or purified virus. Here we used three-dimensional electron microscopy to examine an active infection in the gastrointestinal tract of HIV-1–infected mice with humanized immune systems, allowing visualization of the interplay between the virus and host immune cells. Recapitulating the course of infection in humans, immune cells were depleted in infected humanized mouse gut-associated lymphoid tissue, and individual HIV-1 particles were detected as they budded from host cells and accumulated in pools between cells. HIV-1 was mapped to different substructures and cell types within the gut, and free virions were found to accumulate in pools between cells and also to infect adjacent cells via regions of cell-to-cell contact called virological synapses. Our three-dimensional imaging of an HIV-1 infection in tissue uncovered differences between cultured cell and tissue models of HIV-1 infection and therefore furthered our understanding of HIV-1/AIDS as a disease of mucosal tissues.

## Introduction

HIV-1 remains a significant public health concern with over 33 million people infected world-wide [1]. Most HIV-1 transmissions occur across an epithelial barrier, resulting in generation of a founder population within the mucosa, viral dissemination to lymphatic tissue, and exponential viral replication throughout the lymphatic system [2]. These events result in depletion of most CD4-positive T cells in mucosal compartments, and establishment of a reservoir of resting cells with integrated provirus that is not susceptible to antiretroviral therapy. In the absence of therapy, progressive immune system collapse and progression towards AIDS ensue in most infected persons.

Accumulating evidence indicates that both acute and chronic HIV-1 infection profoundly affect the gastrointestinal (GI) tract [3], [4]. Studies of SIV infection in non-human primates demonstrated that intestinal CD4 T cell depletion occurs within days, even before T cell depletion can be detected in the peripheral blood or lymph nodes [5]; similar events occur in HIV-1–infected humans [2], [6]. Several features of the GI tract facilitate its susceptibility to HIV-1 infection: (i) the GI mucosa includes high levels of pro-inflammatory, HIV-1–stimulatory cytokines produced by exposure to antigens in the external environment, (ii) a dense clustering of cells that facilitates cell-to-cell transmission, and (iii) a majority of the activated memory T cells expressing CD4 and CCR5 that serve as the preferred target cells for HIV-1 infection [7], [8]. Indeed, the gut-associated lymphoid tissue (GALT) harbors the greatest concentration of potential HIV-1 target cells in the human body [9]; >50% of CD4 T cells from the lamina propria in the lower GI tract are destroyed during acute HIV-1 infection, and early infection of the GALT is believed to be central to chronic HIV-1 infection and disease progression [10], [11]. Furthermore, the presence of CD4 and CD8 T cells, dendritic cells, and macrophages in the GALT make this tissue an integral site for HIV-mediated immune depletion.

Mouse models with humanized immune systems are emerging as a tractable, cost-effective means by which to study HIV-1 infection in mucosal lymphoid tissue [12]. One such model, humanized bone marrow/liver/thymus (BLT) mice, are individually created by transferring human fetal thymic and liver organoid tissues, along with CD34-positive human stem cells, into immunocompromised mice. BLT mice reconstitute significant levels of human lymphoid immune cells; e.g., T and B cells, monocytes, dendritic cells and macrophages in peripheral blood and organs including the GI tract [13], [14]. Important aspects of human HIV-1 infection are recapitulated in this system, including T cell depletion in the gut and peripheral blood, and both systemic and mucosal virus transmission during the course of the disease [15], [16]. Furthermore, BLT mice exhibit high levels of human immune cell engraftment at mucosal sites and significant antigen specific immune responses by multiple cell types [17], [18].

Electron microscopy (EM) was instrumental in the original identification of HIV-1 [19], [20]. Subsequently, diagnostic EM analyses of biopsies from infected patients revealed important aspects of HIV-1 transmission in humans at varying stages of infection, from early acute disease to AIDS progression [21]. More recently, 3-D EM, specifically electron tomography (ET), cryoelectron tomography (cryoET) and ion-abrasion scanning electron microscopy, have been applied at increasingly higher resolutions, facilitating improved understanding of HIV-1 virion structure [22]–[24], virus budding [25], [26], and virus transmission between immune cells [27], [28]. 3-D EM of isolated virions and infected cells can provide a detailed understanding of HIV-1 ultrastructure and transmission between cultured cells, but does not address the complex cellular environment found in mucosal tissues within an organism experiencing an active infection.

Here we used ET to analyze GALT from humanized HIV-1–infected BLT mice in order to visualize HIV-1 infection in mucosal tissues in 3-D at ultrastructural resolution. These analyses allowed us to localize infected substructures within intestinal tissue, classify virions as mature or immature, identify infected cells, visualize structures we interpreted as components of the host cell machinery involved in viral budding, and assess the propensity for viral spread by cell-to-cell versus free virus routes of infection. In parallel studies, we used immunofluorescence (IF) and immuno-electron microscopy (immunoEM) to verify the identities of viral particles, locations of infected tissue, and to distinguish human from murine and infected from uninfected cells.

## Results

### Immunofluorescence (IF) Characterization of HIV-1 Infection in BLT GALT

**Figure 1:**
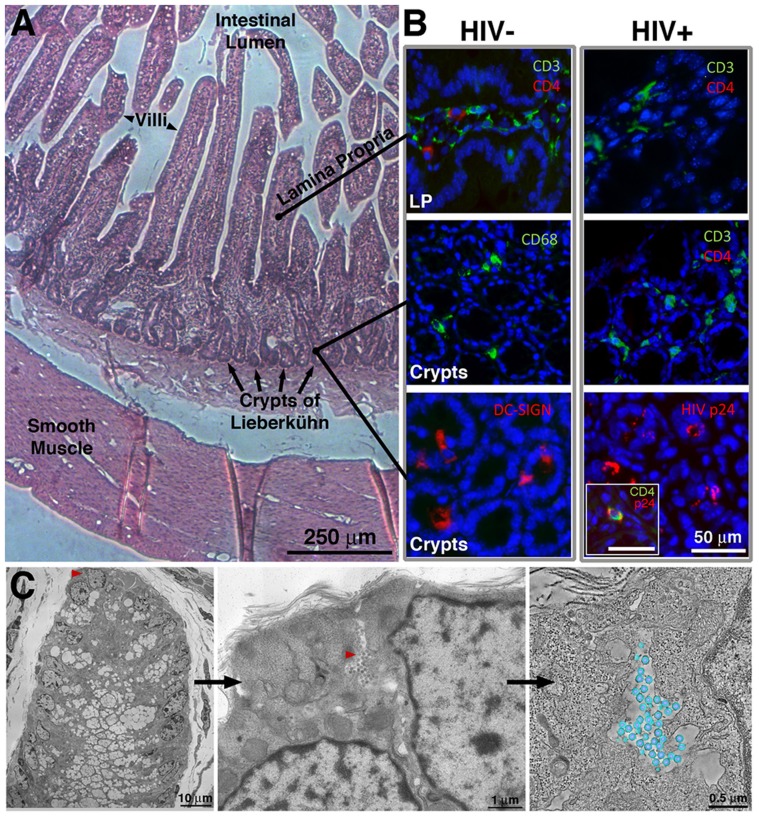
IF and EM imaging of BLT mouse GALT. (A) Histological overview, indicating primary GALT-containing regions. (B) Tissue sections from the small intestine of uninfected or HIV-1–infected humanized BLT mice, stained with antibodies recognizing human CD3 and CD4, CD68, and DC-SIGN (blue = DAPI nuclear stain). The top two panels are longitudinal sections of villi, showing the lamina propria (LP); the bottom four panels are cross-sections showing crypts. Staining for human CD4 revealed depletion of CD4 T cells in both LP and crypts of HIV-1–infected BLT mice, and staining for HIV-1 p24 localized virions to the crypts. Inset: An infected cell co-labeled for CD4 and HIV-1 p24. (C) Left: EM overview of the lower portion of a crypt from BLT-mouse colon. Middle: A pool of free virions (red arrowhead) between two cells. Right: A tomographic slice of the pool with modeled virions (blue, membrane; purple, cores; average diameter = 99.3+/−4.7 nm; n = 50). Figure S1 shows HIV-1 in GALT substructures.

Human hematopoietic cells derived from transplanted human stem cells have been shown to repopulate the GALT of BLT mice, and HIV-1 infection of these mice results in CD4 T cell depletion, initially in GALT and then systemically [13], [16]. Following established protocols [13], BLT mice were infected with HIV-1 approximately 20 weeks after transfer of human immune tissues and cells, using only mice that met the following criteria for adequate human immune reconstitution: >25% of peripheral blood cells were within a lymphocyte gate on forward-versus-side scatter plots; >50% of cells in the lymphocyte gate were human (human CD45^+^/mouse CD45^−^); and >40% of human cells in the lymphocyte gate were T cells (human CD3^+^). Ten to twenty weeks post infection, mice were sacrificed and segments of small intestine and colon were excised. IF was used to survey locations of HIV-1–infected cells in GALT (Figure 1A,B). Following infection with HIV-1, human CD4 T cells were depleted from the lamina propria (Figure 1B), as previously reported [13], [16]. Staining for the p24 capsid protein of HIV-1 localized primarily in CD4+ cells in regions near the crypts (Figure 1B, inset), which harbor significant populations of immune cells and multipotent stem cells [29]. No evidence of human cells or HIV-1 infection was found in non-humanized infected controls (data not shown).

### Ultrastructural Characterization of HIV-1–Infected BLT GALT

We next analyzed GALT samples in parallel by ET and immunoEM/ET. Tomography of frozen hydrated tissue samples by cryoET was not possible because the samples were too thick for imaging without sectioning and were infectious biohazards. We therefore imaged fixed and sectioned samples, either positively-stained plastic-embedded or negatively-stained methylcellulose-embedded sections. For ET alone, preservation quality was improved by lightly fixing HIV-1–infected tissue with aldehydes and then further processing them by high-pressure freezing and freeze substitution fixation [30]. This “hybrid” fixation method allowed for safe handling of infectious material and obviated the most structurally damaging steps of traditional chemical fixation [31], yielding well-preserved positively-stained samples. Tomograms were reconstructed from 200 nm or 300 nm sections, often in montaged serial sections of volumes up to 6.1 µm×6.1 µm×1.2 µm. Although these samples could not be used for immunoEM because antibody epitopes are rarely accessible in epoxy-embedded, positively-stained samples [32], analogous GALT samples generated from the same animal were prepared for immunoEM/ET as negatively-stained methylcellulose-embedded sections [33]. Measurements of virions and other structures reflected proportional thinning typical of plastic-embedded and negatively-stained samples [34]. Consequently most structures were ∼30% smaller than counterparts from cryoEM studies or virions in solution or in cultured cells [22]–[24], [35], [36].

**Figure 2:**
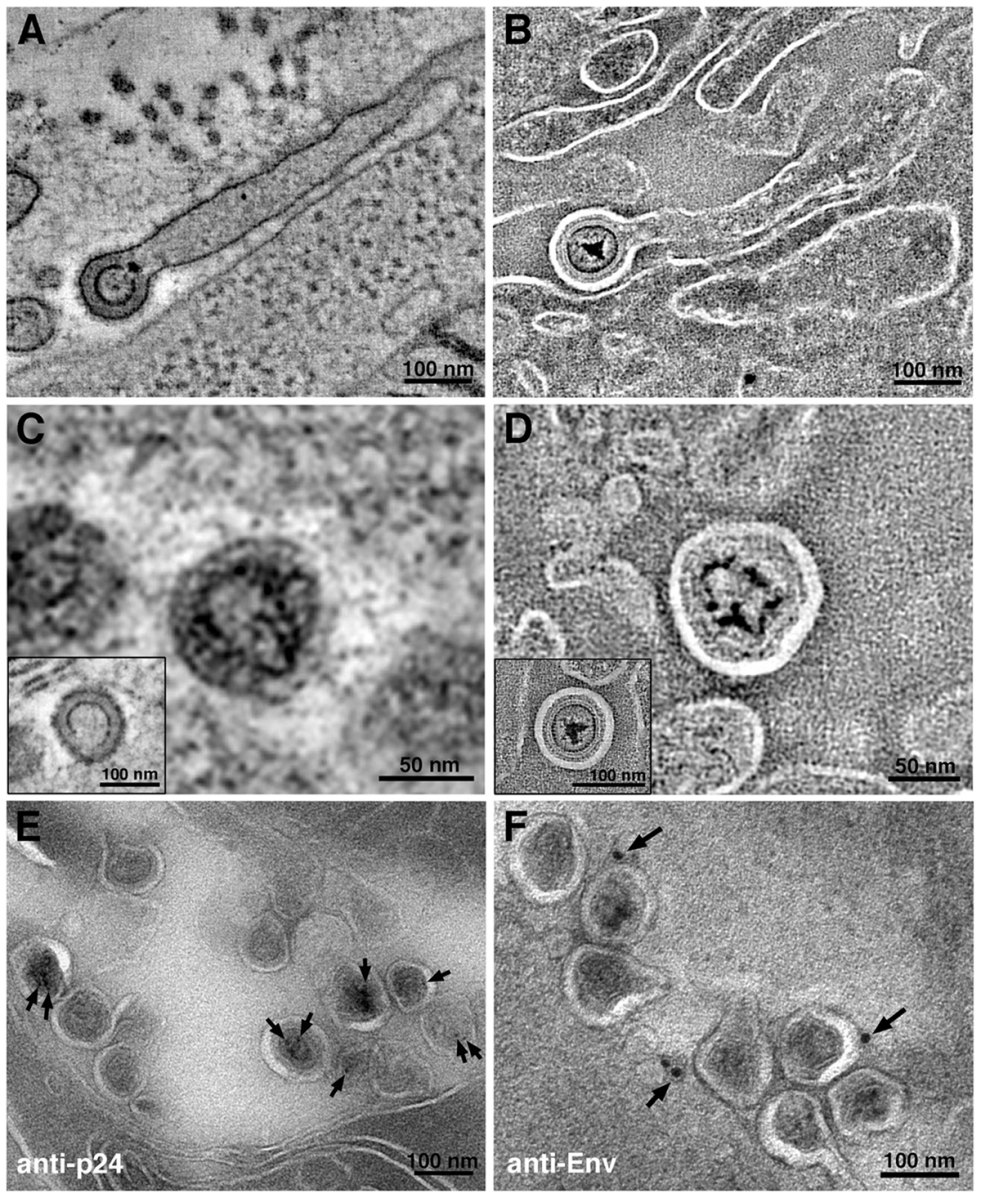
Virion structures in HIV-1–infected GALT. (A, B) Slices from tomographic reconstructions of immature budding virions extending from filopodia in positively-stained (A) and negatively-stained (B) HIV-1–infected jejunum. (C, D) Details from tomographic slices, showing mature and immature (insets) virions in positively-stained (C) and negatively-stained (D) samples. (E, F) Immunolabeling (projection images) of virions labeled with antibodies against the HIV-1 capsid (anti-p24) (E) or the HIV-1 envelope (anti-Env) (F), which localized to the expected regions of the virions: anti-p24 to the interior and anti-Env to the exterior. Figure S3 shows immunoEM of human cell markers.

ET surveys of HIV-1–infected BLT mouse GALT revealed budding virions (Figure 2A,B; Figure S1A) and free mature and immature particles (Figure 1C, Figure 2C–D, Figure S1B). Virions were detected in all HIV-1–infected mice, while none were found in mock-infected controls (data not shown). The virions were verified as HIV-1 using antibodies against HIV-1 p24 and the envelope spike (Figure 2E,F). Virions were imaged in tissue at all stages of egress, from early plasma membrane Gag assembly to nearly completed buds and fully mature, free HIV-1 (Figure S2). Budding profiles and immature free virions were distinguished by core structures that exhibited radial layers and often appeared as an incomplete internal sphere (a “C” shape in projection) [24], [26]. Mature HIV-1 particles were distinguished from immature particles by the collapse of their cores into a variety of conical shapes, typically “bullet-shaped” cones but often cylinders or ellipsoids [23], [37] (Figure S2). Although envelope spikes on HIV-1 and SIV can be distinguished in positively-stained samples [38], we observed few projections emanating from virion surfaces, consistent with biochemical and cryoET studies of purified HIV-1 virions that demonstrated a low number of envelope spikes: an average of ∼14 (ranging from 4–35) per virus particle [39], [40].

After establishing that HIV-1 could be identified in infected BLT GALT by ET and immunoEM, we surveyed GALT samples to determine locations of infection. Plastic-embedded sections of small intestine (jejunum and ileum) and large intestine (colon) were examined to find HIV-1 and infected cells, which were identified by budding profiles at their surfaces. Within a given animal, the extent of infection and the distribution of virions were similar between the small and large intestine. However, virions were found in differing amounts amongst sub-structures in the intestinal mucosa. The largest populations of HIV-1 virions and infected cells identified by EM were located in crypts (Figure 1A,C), consistent with IF (Figure 1B). Approximately one in ten crypts showed evidence of HIV-1 infection. The mucosal region surrounding the villus base and the crypts contained few free virions or infected cells (∼1 in >100); when present, infected cells were often near a capillary or venule (Figure S1A). The numbers of free virions and infected cells in the lamina propria were less than in the crypts (Figure S1B). Typically, infected lamina propria were in villi continuous with infected crypts. Few infected cells or virions were found in the smooth muscle layer surrounding the intestine. In addition, free virions were rarely found in blood vessels because even the high viral loads of the HIV-1–infected BLT mice from which the samples were derived (up to 126,000/mL in peripheral blood) translated to only ∼1×10^−7^ virions/µm^3^. Thus at the scale of individual EM images or even large-format tomograms, HIV-1 virions would be rarely seen, and our imaging of >50 blood vessels contained within tomograms yielded only two examples of free virions (data not shown).

To identify potential human target cells of HIV-1 infection, we conducted immunoEM (Figure S3A–D) using antibodies specific for human proteins. Human CD4 localized primarily to the plasma membrane in uninfected cells (Figure S3A), but we found extensive CD4 labeling in the endoplasmic reticulum (ER) of CD4-positive cells with budding virions or nearby free virions (Figure S3B), correlating with the finding that HIV-1 Vpu induces cell surface CD4 to redistribute to the ER to avoid surface retention of newly-forming virions [41]. Double labeling with antibodies against HIV-1 Nef and human CD4 (Figure S3C) or class I human leukocyte antigen (HLA) and human CD4 (Figure S3D) confirmed that cells exhibiting a predominantly ER localization of CD4 were human cells infected with HIV-1. No instances of Nef expression were found in uninfected or non-human cells (data not shown), which served as an internal control for the specific of the antibodies and further validated the BLT model of HIV-1 infection.

### Structural Details of Immature HIV-1 Virions in GALT

**Figure 3:**
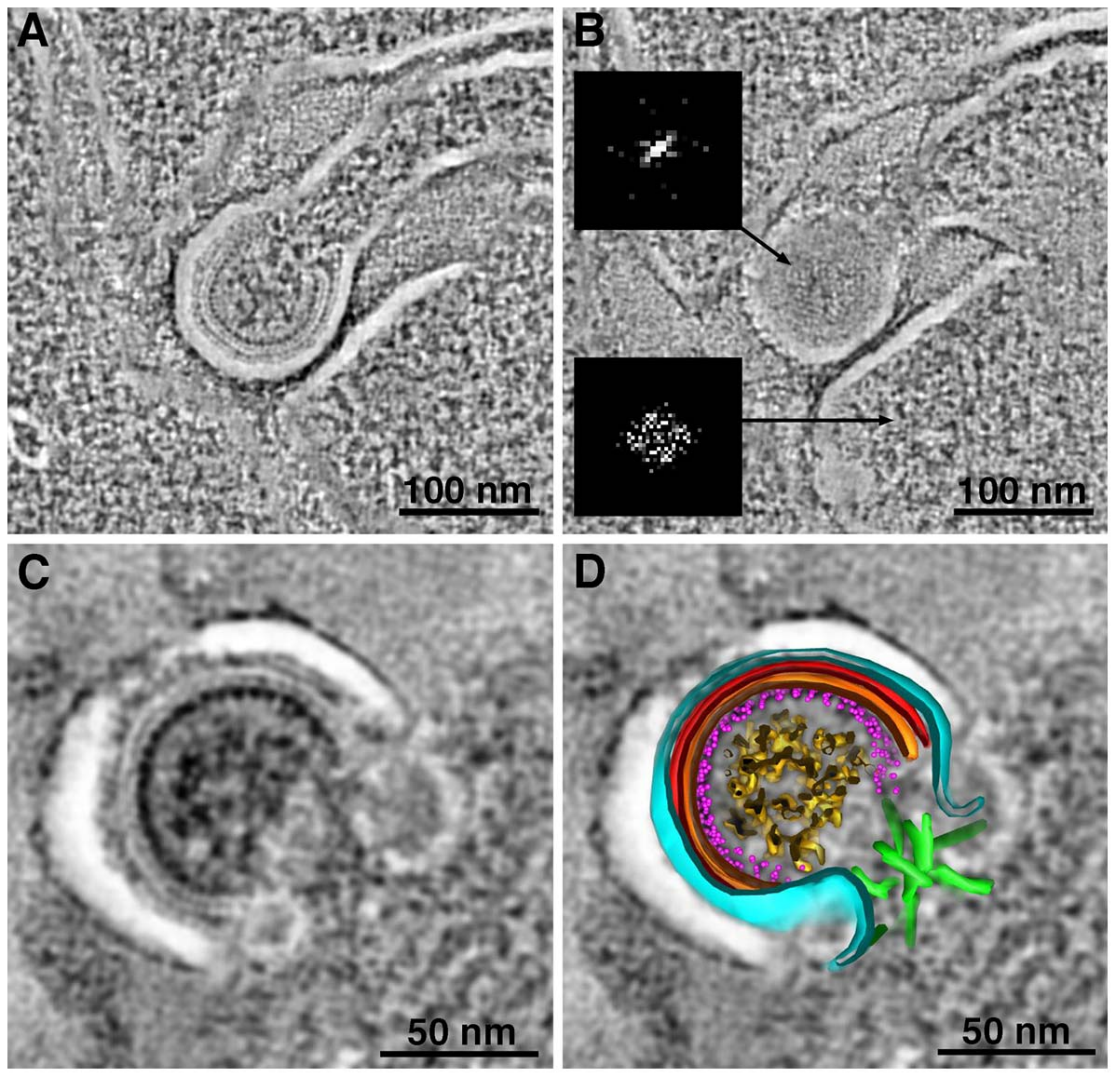
Structural details in negatively-stained images of HIV-1 in infected GALT. (A, B) Tomographic reconstructions of budding HIV-1 virions, showing Gag layers (A; slice through equator) and hexagonal lattice (B; slice through surface). Hexagonal symmetry was confirmed by Fourier transformation of the Gag lattice region in a single tomographic slice (upper inset in B). A similar transform of a region of cytoplasm adjacent to the bud (lower inset) shows only the inherent Friedel symmetry of a Fourier transform. (C, D) Tomographic slice (C) and model (D) of a budding profile. The identities of the layers in the budding profile cannot be definitively assigned, but a proposed assignment of features visible in tomogram is as follows: black, plasma membrane; light blue, MA; red, CA-NTD; orange, CA-CTD; magenta dots, NC; gold, RNA genome; green, ESCRT. The white space between the plasma membrane and first Gag layer (see also panels A–C) is an artifact of preservation. Figure S4 shows a gallery of Fourier transforms and a comparison with cryoET.

Tomograms of immature virions derived from negatively-stained infected tissue revealed detailed structural information. With the exception of the widening of lipid bilayer membranes, presumably caused by obligatory light fixation associated with this method, the overall architecture of the Gag shell in immature virions conformed to known properties of HIV-1 determined from studies of viruses isolated from cultured cells [22], [24], [35], [36], [42] (Figure 3; Figure S4). Indeed, the immature virions in our tissue samples (Figure 3, S4A,B) exhibited features observed in cryoET analyses of purified frozen hydrated HIV-1 [22], [24] (Figure S4C); e.g., individual layers of the Gag shell, including the hexagonal lattice of the capsid (CA) portion (Figure 3). The symmetry of the CA layer was confirmed by hexagonal features in the Fourier transforms of immature virions, but not in transforms of adjacent cytoplasm (Figure 3B; Figure S4A).

### Identification and Quantification of Intercellular Pools of HIV-1 Virions

**Figure 4:**
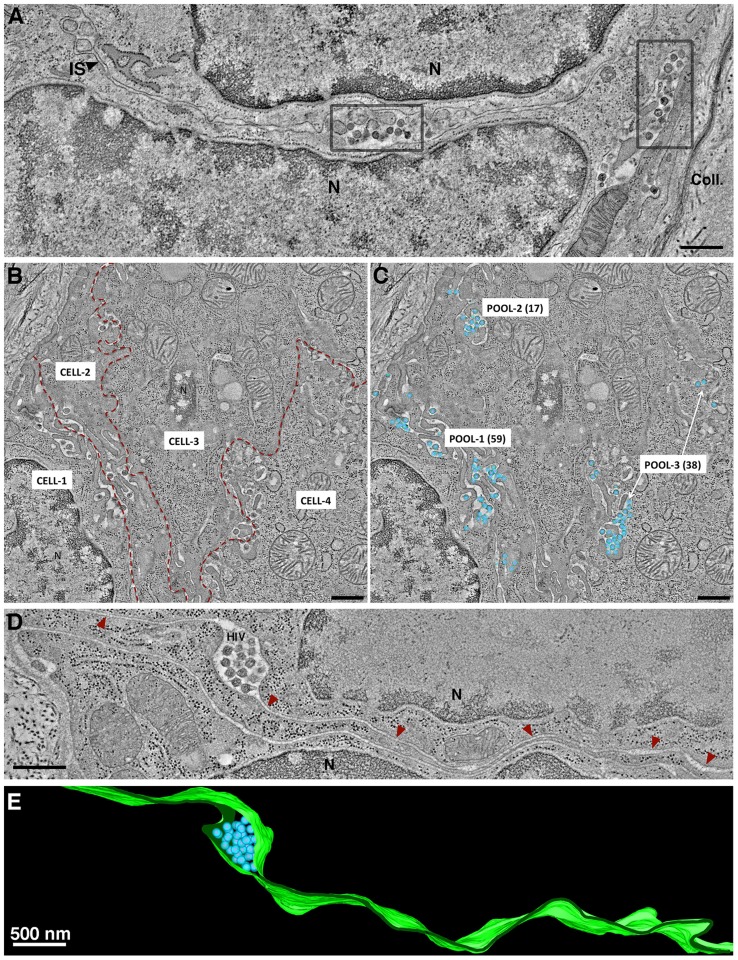
Intercellular pools of HIV-1. (A) Tomographic slice of a GALT region near the edge of a crypt. Two pools of mature HIV-1 virions, indicated in gray boxes, occupied dilated regions of the intercellular space (IS) between two cells (N, nucleus). Collagen fibrils (Coll) were visible at the outer boundary of the crypt. (B) Montaged overview of four cells within GALT. Red dashed lines demark the intercellular spaces. Pools of virions within GALT were defined as a population within an intercellular space that was continuous throughout a given volume of a tomographic reconstruction. Virus pools within intercellular spaces that did not connect within the volume were considered distinct. (C) Region shown in B with modeled HIV virions in three pools containing 59, 17 and 38 virions, respectively. (D) Pool of HIV-1 in a dilated domain associated with a thin channel (red arrowheads) that opened to the mucosa. (E) Segmented model of the microchannel shown in D. The width of the channel remained relatively constant through an ∼600 nm volume, suggesting that morphological changes would be necessary for virions to escape. (A: jejunum; B–E; colon).

More than 50 crypts of Lieberkühn were imaged in the course of this study. In the ∼10% of crypts that were infected, HIV-1 virions were found primarily in pools within dilated regions of intercellular spaces (Figure 1C; Figure 4; Figure S5; Movie S1). Pools were defined as a population of virions within an intercellular space that was continuous within a given 3-D volume. Multiple intercellular spaces could be present within the volume, but unless the spaces were visually continuous, virions within them were regarded as separate pools (Figure 4B,C). The numbers of free virions in intercellular pools ranged from 5 to >200. In single-frame tomograms (3.2 µm×3.2 µm×200 nm), most pools contained 10–40 particles. Larger pools were observed in serial-section reconstructions encompassing greater tissue volumes. In longitudinal sections of crypts, most pools were found between the base and middle. Infected human immune cells, identified by the presence of budding virions, were often found near virion pools.

Virions within a given pool were distinguished as mature or immature based on the presence of a cone-shaped core in mature particles and radial Gag layers in immature particles (Figure S2). The numbers of mature and immature particles in intercellular pools were quantified within reconstructed volumes of infected crypts. Pools could be classified as either “mostly mature” or “mostly immature” (Figure S5A). Of >100 pools containing many hundreds of virions, approximately 90% of pools were classified as mostly mature and 10% were mostly immature.

Potential HIV-1 target cells and pools of virions were plentiful in GALT, particularly in crypts, thus it was not always possible to determine from which cell a particular virion population originated. In order to quantify virions from a particular cell and infer temporal data with respect to virion pools, we imaged regions of the intestinal smooth muscle layer (Figure 1A), which contains few HIV-1 target cells. Figure S5B shows an HIV-1-infected cell in the smooth muscle. The surface of this cell exhibited several HIV-1 budding profiles, and groups of free virions were located both in close proximity to and at varying distances from it. There were no other infected cells within several microns, thus we could be confident that nearby free virions had originated from that cell. We found that 62% of virions (n = 16) in immediate proximity (≤0.5 µm) to the cell were immature, while 73–75% of virions in groups located 0.8 µm (n = 15) and 1.3 µm (n = 32) away were mature.

Of >100 virion pools that were imaged, most were in obvious extracellular spaces. Some pools (∼5%) appeared to be intracellular, but were revealed by ET to be connected to the extracellular space by narrow channels that averaged ∼27 nm in width (range = 23–32 nm; n = 6) (Figure 4D–E) and contained 2–20 mature virions. A few of the budding regions were large enough that potential continuities with the plasma membrane were outside of the reconstructed volume. The presence of seemingly intracellular virion pools connected to microchannels could identify the cell as an infected macrophage, a cell type in which internal virus-containing compartments were proposed to represent specialized domains of the plasma membrane that were sequestered intracellularly [43], [44] and/or endosomal compartments [45], [46].

### Pools of Free Virions Versus Cell-to-Cell Transmission of HIV-1

**Figure 5:**
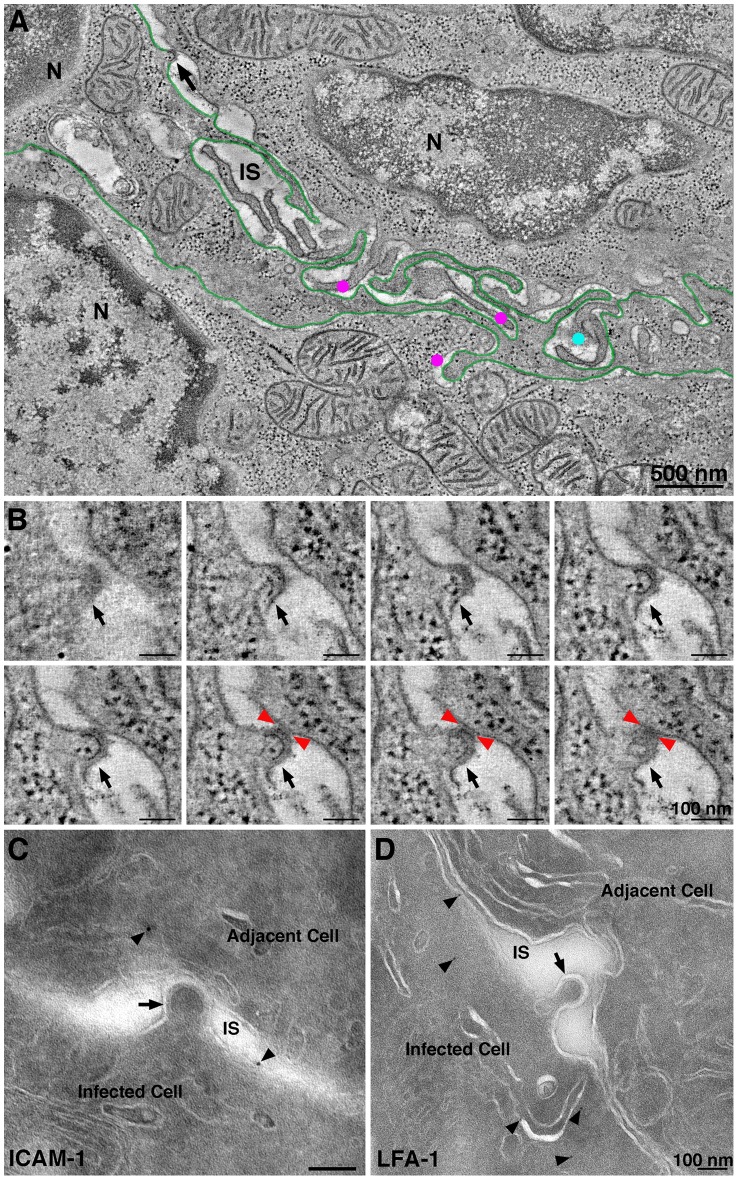
Intercalating infected cell with a budding profile contacting an adjacent cell. (A) Tomographic slice (9 nm) from a six-frame montaged tomogram near the edge of a crypt in colon. The field contained two cells (N, nucleus) with an intercalating HIV-1–infected cell (presumably a dendritic cell; green outline). Four HIV-1 budding profiles were forming from the presumptive dendritic cell at different positions in the volume (magenta dots and black arrow). Dots indicate the approximate position of free mature virions at different positions in the volume (upper cell: light blue; lower cell magenta); the black arrow indicates a budding profile potentially involved in a virological synapse. (B) Eight tomographic slices (9 nm each) detailing the approach of the bud in panel A (black arrows) to the adjacent cell. Red arrowheads indicate the points of contact with the adjacent cell. (C) Immunolocalization of ICAM-1 near a presumptive virological synapse. A budding virion (arrow) is shown projecting from an infected cell and contacting the surface of an adjacent cell across an intercellular space (IS). The surfaces of both cells labeled for ICAM-1 (arrowheads). (D) Immunolocalization of LFA-1 near a presumptive forming virological synapse. The budding profile (arrow) extending from the infected cell was nearing the surface of the adjacent cell. LFA-1 (arrowheads) was present on the surface and proximal underlying compartments of the infected cell. Details of this interaction are shown in the latter part of Movie S1. Other examples of potential virological synapses are shown in Figure S6.

ET surveys of HIV-1 infected GALT showed evidence of virological synapses for direct cell-to-cell virus transmission, a route of HIV-1 transmission within tissues whereby a virus buds from an infected cell and directly contacts and infects an adjacent uninfected cell [47]. Formation of a virological synapse results from interaction of gp120 on an infected cell with its receptors on a target and also involves other host proteins such as LFA-1 and ICAM proteins on the surfaces of both the donor and target cells [48], [49]. A large format reconstruction (2×3-frame montage) of GALT revealed an HIV-1–infected cell, likely a dendritic cell or macrophage based on the convoluted processes intercalating between neighboring cells (Figure 5A; Movie S2). A presumptive virological synapse was visualized as a region of contact between a budding virion and an adjacent cell (Figure 5B; Movie S2). Although this positively-stained sample could not be examined by immunoEM, we found similar features in negatively-stained samples that labeled with antibodies against LFA-1 and ICAM-1 (Figure 5C,D), supporting the identification of these regions as virological synapses. In another example, an infected cell that showed numerous budding profiles included one that closely approached the surface of an adjacent cell although still attached to its host cell via a ∼50 nm neck (Figure S6A). The surface region of the cell proximal to the approaching bud was denser than surrounding surface regions and extended toward the bud. In a third example, a budding profile from an infected cell appeared to project into an invagination in the plasma membrane of an adjacent cell (Figure S6B,C). Tomographic views through the volume containing this region showed the boundaries of the invagination followed the contours of the budding profile (Figure S6C), suggesting a dynamic response to the approaching nascent virion.

By reconstructing a large 3-D volume of infected tissue, we could address whether direct cell-to-cell transmission was an obligatory means of virion transfer between two adjacent cells. Movie S1 shows a 1.4 µm×2.9 µm×1.2 µm tomogram in which the outlines of two adjacent cells were distinguished. Both cells were identified as infected by the presence of budding virions and were therefore HIV-1 targets. A region resembling a virological synapse was not observed in the reconstructed volume, however a large accumulation of free mature virions were present in the space between the cells, suggesting that direct cell transfer is not a required mechanism of HIV-1 transmission between closely apposed infected cells. The lack of an observed virological synapse in such cases could be the consequence of CD4 down-regulation in the infected cells. However the existence of natural recombinant HIV-1 strains, which could result from infection by one HIV-1 strain of a cell already infected with a different viral strain [50], suggests that residual CD4 remaining at an infected cell surface can allow for infection via free virus or direct cell-to-cell transfer.

### Characteristics of Budding Virions

The large number of budding virions within BLT GALT tomograms offered the opportunity to characterize structural aspects of HIV-1 budding in infected tissue (Figure S7). Actin filaments were often found near forming buds (Figure S7A) similar to those previously observed at HIV-1 budding sites in cultured cells [25]. Budding profiles exhibited varying lengths of necks, including some with no neck (Figure 3C,D; Figure S7B). In the colon, early budding virions without necks were often observed forming from surfaces that were not obviously plasma membrane. However, serial-section tomography revealed that these domains were usually continuous with the plasma membrane proper, indicating that they were convoluted regions of the cell surface and not distinct cytoplasmic compartments. Some budding virions exhibited necks with 50–80 nm lengths and varying widths (Figure S7C), with narrower necks likely representing those approaching scission. Virions were also observed budding at the ends of extremely long cellular projections (Figure 2A,B) that were likely filopodia extending from dendritic cells, as observed in culture [27], [51].

ET analyses of HIV-1 budding in cultured cells revealed a subset of RNA-free immature virions with a novel “thinner” Gag lattice lacking the nucleocapsid-RNA layer, which were suggested to represent aberrant, noninfectious virions resulting from premature activation of HIV-1 protease [25]. Using our measuring convention, the previously-described thin Gag lattice [25] measured 9–10 nm. Analysis of 100 free or budding immature virions from tissue samples yielded no examples with a thin (9–10 nm) Gag lattice that lacked discernable RNA densities. Instead, we found that the Gag lattice widths in all of the immature virions we surveyed (n = 100) within infected tissue was 14.6±0.8 nm (Figure S4B, Figure S7D,E); significantly different than the thin 9–10 nm Gag lattices previously described [25]. In addition, there were no systematic structural differences in Gag lattices correlating with the type of budding profile: the Gag shell thicknesses measured in 30 long-necked and 30 neck-free buds were similar and presumptive RNA densities were present in all cases (Figure S7D).

### Localization of ESCRT Pathway Components at Sites of HIV-1 Budding

Release of HIV-1 virions from infected cells involves recruitment of the host endosomal sorting complexes required for transport (ESCRT) machinery to sites of virus assembly by the Gag polyprotein [52]. These interactions culminate with the polymerization of ESCRT-III proteins, recruitment of vacuolar protein sorting-associated protein 4 (VPS4) ATPase oligomers, fission of the cellular membrane attaching the virion to the host cell, and disassembly of the ESCRT machinery.

**Figure 6:**
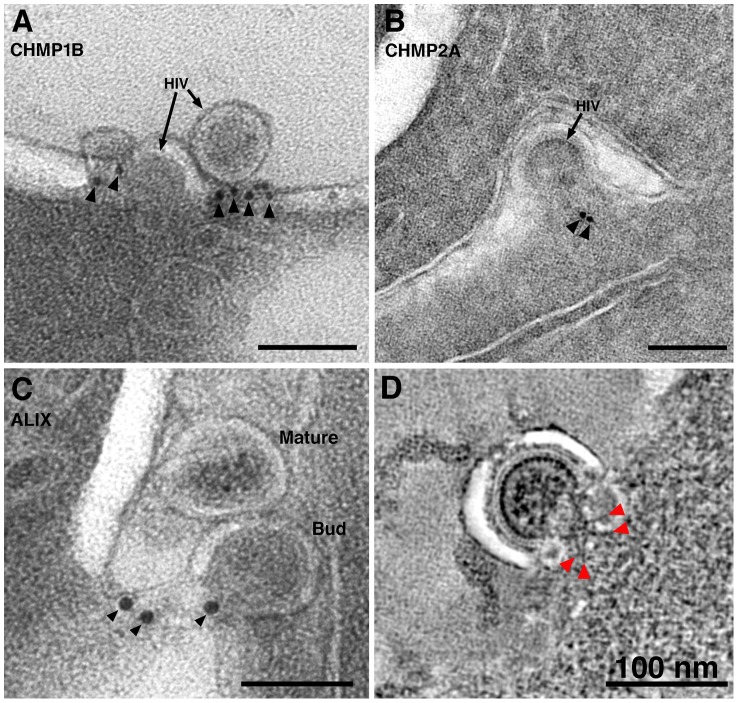
ImmunoEM of ESCRT pathway proteins at sites of HIV-1 budding in GALT. (A–C) Immunolabeling (projection images) of budding virions using antibodies against CHMP1B (A), CHMP2A (B), and ALIX (C). Antibodies localized to the necks of budding virions or to the adjacent plasma membrane. (D) Cluster of spoke-like projections (red arrowheads) radiating from a common origin (see also Figure 3C,D). These types of striations, suggested to represent components of ESCRT-I and/or ESCRT-II, were only seen when the neck diameter was more than half of the diameter of the bud. Figure S8 shows galleries of electron dense structures from both “early” and “late” budding HIV-1 virions.

We used antibodies against ESCRT-III proteins, human charged multivesicular body proteins (hCHMPs) 1B and 2A, and ALG2-interacting protein X (ALIX), an ESCRT adaptor protein that facilitates the transport of Gag to the cell membrane [53] and can mediate interactions between ESCRT-I and ESCRT-III complexes [54], to detect components of the ESCRT pathway in infected tissue by immunoEM. We found that hCHMP1B, hCHMP2A and hALIX localized predominantly to the neck regions of budding HIV-1 virions (Figure 6A-C). The labeling was specific, but sparse due to the small number of epitopes and their availability only at section surfaces.

**Figure 7:**
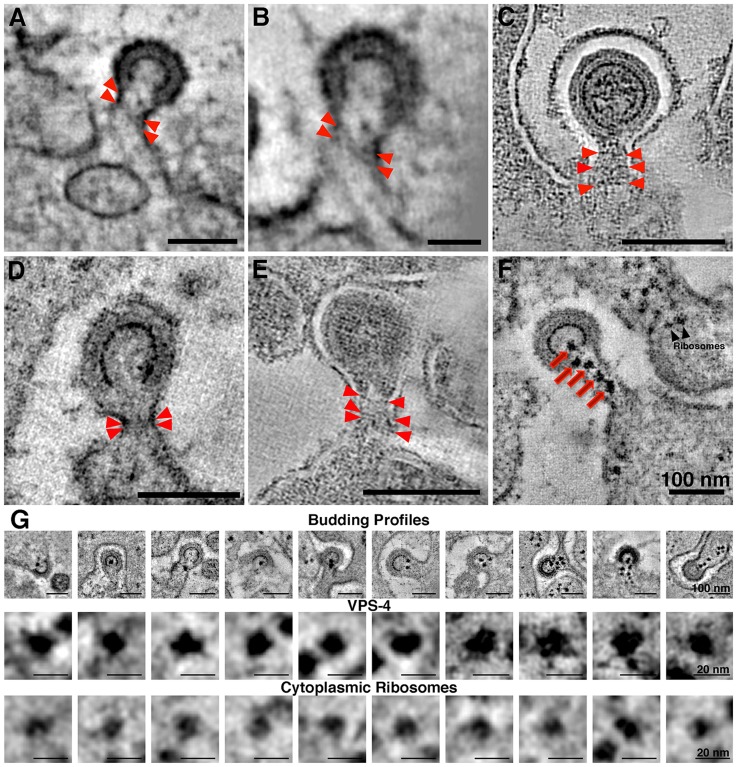
Electron dense striations in tomograms of the scission regions of budding virions. (A–E) Parallel electron dense striations (red arrowheads) circumscribing the necks of budding virions in positively-stained, plastic-embedded samples (A,B,D) and in negatively-stained samples (C,E). Parallel striations, suggested to be portions of the polymerized ESCRT-III complex, were observed only when the diameter of the neck was half or less than the diameter of the bud. (B) Higher magnification view of the bud in (A), rotated to optimize visualization of the striations. (F) Tomographic slice of a positively-stained budding profile displaying five dense spots (red arrows) in the neck region that may correspond to VPS4 complexes recruited to facilitate scission of the bud. Ribosomes in cytoplasm distal from the budding virion appeared slightly smaller and typically less electron dense than the presumptive VPS4 structures. (G) Galleries of HIV-1 budding profiles bearing presumptive VPS4 spots (row 1) and individual presumptive VPS4 spots and cytoplasmic ribosomes, extracted from tomograms and viewed at high magnification (rows 2 and 3, respectively). The VPS4 spots were pleomorphic and solidly dense, with an average width of 13.3±0.8 nm; n = 10). The spots appeared to be slightly larger than ribosomal densities (11.9±0.8 nm; n = 10), which were less dense and often showed a characteristic “groove” between the 30S and 50S subunits. Note that the ribosomes in this and previous ET studies involving positively-stained, plastic embedded samples [72] appear smaller than their 25–30 nm diameter.

At scission regions of budding virions in which the neck of the bud was greater than half the diameter of the bud, clusters of 4–6 spoke-like projections nearly 20 nm in length radiating from a centralized origin at the base of the budding virion were sometimes observed (Figure 3C,D; Figure 6D; Figure S8A; Movie S3). As the larger neck diameter may define these buds as being at an initial stage of egress, these radial projections could represent components of the early portions of the ESCRT pathway such ESCRT-I or ALIX recruited by assembling HIV-1 Gag molecules. Indeed, the size and shape of the structures approximate models for the ESCRT-I-II supercomplex determined by a combination of spectral techniques [55]. By contrast, in tomograms of budding virions with narrower necks (less than half the diameter of the bud itself), we observed parallel electron dense striations circumscribing the neck of the bud in both positively- and negatively-stained sections (Figure 7A–E; Figure S8B,C; Movie S4) suggestive of ESCRT-III components polymerizing at membranes [56], [57]. Similar electron dense striations were detected at the necks of budding virions arrested at a late stage by expression of dominant-negative ESCRT-III or VPS4 proteins [58]. In addition, budding profiles in positively-stained samples often showed 1–5 electron-dense “spots” in the neck or base of a bud (Figure 7F,G; Movie S5). The spots were observed in over half of ∼50 budding profiles in which the diameter of the neck was half or less of the diameter of the budding virion; presumably a late stage of budding. Available antibodies against VPS4 did not stain efficiently by immunoEM, however their interpretation as VPS4 oligomers was consistent with fluorescence imaging showing recruitment of 2–5 VPS4 dodecamers to the sites of viral budding just prior to virion abscission [59], [60]. In addition, the size and relative shape of the putative VPS4 densities (Figure 7G) correlated with cryoEM reconstructions of VPS4 [61].

## Discussion

Many aspects of the pathologies related to HIV-1 infection, including immune cell death and tissue destruction, occur in GALT. However, 3-D ultrastructural details of a natural GALT infection were unknown because ET had not been applied to in vivo infection in GALT or other lymphatic tissues. BLT humanized mice are an emerging model for studying HIV-1 infection, and BLT GALT maintains cellular architecture, cell-cell interactions, immune cell populations and signaling more accurately than cell culture infection models [12]. As such, the BLT mouse system is a reliable model for structural studies of HIV-1 infection in a tissue environment. In addition, the inclusion of human thymic tissue in BLT mice allows for T cell maturation in the context of human, rather than murine, MHC proteins; an aspect that is not present in humanized mouse model systems produced with human hematopoietic stem cells but without thymic tissue.

Dense areas of HIV-1–infected cells, including CD4 T cells, macrophages and dendritic cells, and free HIV-1 virions were found in crypts within BLT GALT by IF, ET and immunoEM (Figure 1B,C). Blood vessels were imaged in mice with a wide range of viral loads; however, we were unable to correlate the relative abundance of virions detected in GALT with the viral load measured in the blood. In fact, only two examples of virions within blood vessels of BLT mice were detected as compared with hundreds of virions within mucosal tissue. This finding is consistent with reports highlighting a discrepancy between blood viral load and HIV-1 levels in tissues [62], [63]. Thus analysis of HIV-1–infected tissues by methods such as ET may provide valuable information in addition to blood viral load measurements when evaluating treatment regimens.

Potentially relevant to infection and immune cell recognition mechanisms, large pools of free HIV-1 were found within infected GALT (Figures 1C, Figure 4, Figure S5). Although most pools contained mainly mature virions, some pools contained a majority of immature virions (Figure S5A), a phenomenon not observed in EM studies of HIV-1 infection of cultured cells. Pools of virions were usually found between cells, but also in compartments that appeared to reside within cells. These compartments were often connected to the cell surface by microchannels 20–30 µm in width (Figure 4D). These narrow channels likely undergo dynamic changes in morphology, as their width would be too narrow to accommodate passage of HIV-1 to the extracellular space. We interpreted such channels as invaginations of the plasma membrane, consistent with reports that macrophages can assemble HIV-1 in intracellular virus-containing compartments created by internally sequestered plasma membrane [43], [44], [64]. In infected tissue, we found that pools of HIV-1 virions located between two cells could contain mature or immature virions (Figure S5A), whereas the intracellular pools connected by microchannels contained only mature virions (Figure 4D). One possibility for the difference in maturation states of inter- versus intracellular pools of HIV-1 is that intracellular virions connected to the extracellular space by microchannels are not subject to movement by interstitial fluid through intestinal tissue and could remain in a single location long enough to complete maturation, perhaps representing viral reservoirs that allow low levels of de novo infection to proceed in the presence of anti-retroviral therapy and/or antibodies [65].

Although the discovery of virion pools suggested that infection by free virus could occur within infected tissue, we also found evidence of direct cell-to-cell transmission of HIV-1 in infected GALT (Figure 5; Movie S2). The virological synapse is a mechanism of cell-to-cell transmission in which juxtaposition of an infected and uninfected cell promotes infection by directing viral assembly, budding, maturation, and fusion machinery to discrete locations of cellular contact between cells [47]. In a large 3-D reconstruction of two adjacent HIV-1–infectable target cells (Movie S1), we found a large pool of mature virions but no evidence for a virological synapse, suggesting that formation of virion pools and infection by free virus can occur even when adjacent cells are both infectable by HIV-1, or had been infectable prior to down-regulation of CD4. In addition, this result validated our frequent finding of large pools of free virions in HIV-1–infected tissue, demonstrating that this phenomenon was not necessarily the consequence of the juxtaposition of a human infected cell and a murine cell, as may occur in BLT GALT.

EM studies of HIV-1 virions produced in cultured cells suggested that maturation is a rapid process, because intermediate maturation states were not detected and because virions found near cells were predominantly mature [66]. However, our finding of pools containing immature virions in proximity to infected cells in tissue suggested maturation dynamics and/or virion diffusion properties differ between cells organized within tissue versus those cultured in vitro. In addition, we never found examples of RNA-negative budding virions with a thin Gag lattice in tissue samples, as had been observed in ∼18% of immature particles in cryoET analyses of HIV-1 produced in cultured cells [25]. Thus, higher numbers of aberrant particles and of exclusively mature virions in close proximity to producer cells could be artifacts of producing virions in cultured cells, suggesting that the BLT model of in vivo infection more accurately recapitulates the HIV-1 lifecycle than cell culture models.

Although ET relies on fixed tissue and cannot directly recapitulate virion dynamics in live cells, our studies provided a glimpse into temporal aspects of HIV-1 maturation. We determined that an isolated infected cell within a large tissue volume was the sole producer of several populations of imaged virions located at varying distances from the cell. This allowed us to determine that a single infected cell can produce at least 63 viruses (the number of virions in the three pools in Figure S5B). The total number of virions produced per cell is likely far larger, as regions above and below the cell were not represented in the reconstruction. Using a predicted rate of interstitial fluid movement in intestinal tissue of 0.1–2 µm/sec [67], a virion would travel 2 µm in 1–20 sec, indicating that maturation could occur just seconds after release from an infected cell. This argues that, in tissue, virions found ∼2 µm away from a producer cell budded only seconds earlier, supporting an assumption of rapid virus maturation. Furthermore, our finding of mostly immature virion pools in close proximity to the infected cell and mostly mature virion pools further away from the cell (Figure S5B) is consistent with synchronous release and subsequent maturation of HIV-1. The trigger(s) for and/or block(s) to maturation that could promote synchronized virus maturation in tissue could include proximity to an infected producer cell, lack of an adjacent target cell to form a virus synapse, and/or contact with a non-infectable cell.

Late events in HIV-1 budding had been visualized by fluorescence microscopy [59], [60] and ET of cultured cells [25], [26], [66], [68] but not yet in infected tissue. In our infected tissue samples, we detected distinct electron dense structures near virions at various stages of budding that may represent aspects of the host cell ESCRT machinery at sites of HIV-1 egress (Figures 6,7,S8). Although we could not identify the structures conclusively, our assignments of their possible identities are consistent with what is known temporally about the involvement of host cell machinery in HIV-1 budding and release from infected cells. Tomograms revealed that virions in the initial stages of budding contained 4–6 spoke-like projections emanating from the center of the forming neck of the budding virion (Figure 3C–D, Figure 6D, Figure S8A), potentially representing components of host ESCRT-I or ALIX recruited by assembled HIV-1 Gag. The shape, size, and temporal occurrence of these structures agree with a proposed model for vesicle budding and fission based on biophysical analyses of the ESCRT-I-II supercomplex in solution [55]. Virions at later stages of budding that were connected to the host cell membrane by thinner (<50 nm) elongated necks showed parallel, electron dense striations along the membrane surface of the neck (Figure 7A–E, Figure S8B) that we interpreted as features of polymerized ESCRT-III proteins [56], [57]. These late budding profiles often displayed dense spots along the center of the neck (Figure 7F,G) that we suggest were VPS4 oligomers recruited immediately prior to fission of the new virion from the cell membrane, consistent with fluorescence microscopy studies [59], [60]. ET of budding virions within tissue allowed a spatial and temporal interpretation of HIV-1 budding. First, the Gag lattice reached a sufficient point of closure, which allowed formation of a spoke-like structure at the base of the early budding virion. Next, the virion formed an elongated neck; concomitant with polymerization of host cell factors in a spiral around the inside of the membrane [56], [57]. In tomographic slices of budding profiles, these presumptive spirals appeared as two or more parallel lines bisecting the neck region (Figure 7A–E). Finally, the recruitment of large oligomers, possibly VPS4, coincided with the separation of the virion from the infected cell [59], [60], completing the budding process (Figure 7F,G).

In summary, our 3-D ultrastructural characterization of HIV-1–infected GALT identified dense regions of virus transmission, provided insights into the temporal nature of virus maturation, revealed HIV-1 transmission occurring by both free virus and direct cell-to-cell mechanisms, and demonstrated important differences between cultured cell and tissue HIV-1 infection models. Differences included the identification of free immature virions and the scarcity of aberrantly formed viral particles during an active infection. The high resolution of our positively- and negatively-stained tissue samples allowed 3-D visualization of HIV-1 transmission within lymphoid tissue, providing a new approach for understanding HIV-1 infection in vivo.

## Materials and Methods

### HIV-1 Infection of BLT Mice

Humanized mice were prepared and cared for in an AAALAC-certified animal care facility at the Massachusetts General Hospital (OLAW Assurance #A3596-01), in accordance with a protocol approved by the MGH IACUC (Protocol #2009N000136/25). The protocol as submitted and reviewed conforms to the USDA Animal Welfare Act, PHS Policy on Humane Care and Use of Laboratory Animals, the “ILAR Guide for the Care and Use of Laboratory Animals” and other applicable laws and regulations. Every effort was made to minimize animal suffering throughout all experiments. Human tissue for preparing the humanized mice was procured and used in accordance with a protocol approved by the local Institutional Review Board (Partners Human Research Committee, Protocol #2012-P-000409/5).

NOD/SCID/IL2Rγ−/− mice (The Jackson Laboratory) were reconstituted with human tissue as described [13]. Approximately 20 weeks after transfer of human immune tissues and cells, mice were infected intraperitoneally with 1×10^5^ TCID_50_ of JR-CSF HIV-1. Every 2 weeks after infection, ∼200 µl of blood was obtained through puncture of the retro-orbital sinus or submandibular vein for determination of HIV-1 plasma viral load. Viral RNA was isolated using the QIAamp Viral RNA Mini Kit (Qiagen) and viral loads were determined by quantitative RT-PCR using primers for HIV-1 Gag [69]. Immunofluorescence experiments were conducted using tissues from a mouse with a blood viral load of 940,000 copies/mL. ImmunoEM experiments were conducted using tissues from a mouse with a viral load of 100,000/mL. The remaining mice had blood viral loads as follows: 0 (control) 9,800, 8,400, 18,500 and 126,000 copies/mL. As previously shown, the range of blood viral loads did not correlate with virus populations found in tissue samples [62], [63]. Infected mice were sacrificed 10–20 weeks post infection, and then necropsied with segments of small intestine and colon excised and fixed. Immunofluorescence (IF) studies were conducted as described in the Supplementary Methods (Text S1).

### Electron Tomography

For positively-stained samples, HIV-1–infected tissue was prepared by a hybrid method that employed primary chemical fixation followed by high-pressure freezing/freeze substitution fixation (see Text S1). Negatively-stained samples were prepared as described [33] and in the Supplementary Methods (Text S1).

200 nm positively-stained sections and 90 nm negatively-stained sections were imaged in a Tecnai-12 G2 transmission electron microscope at 120 KeV, and 300 nm sections were imaged in a Tecnai G2 TF30-FEG microscope at 300 KeV (FEI Company, Holland) in a dual-axis tomography holder (2040; Fischione Instruments, Export, PA). Dual axis tilt series (+/−60°; 1° intervals), including multi-frame montaged datasets, were acquired automatically using the SerialEM software package [70]. Tomographic data were aligned, backprojected, analyzed and segmented using IMOD [71].

### Gag Lattice Analyses

The Gag lattice in the tomographic slice closest to the equator of each virion or budding profile slice was measured in five randomly selected areas as a line from the base of the innermost layer to the outside of the outermost layer (green lines in Figures S3B and S6D) using IMOD [71]. The values were combined to give an average Gag thickness for each virion. The symmetry of the Gag lattice was evaluated by Fourier transformation of Gag regions in negatively-stained tomograms. Budding profiles were viewed in tomographic slices taken near the surfaces of their Gag layers (Figure 3B) and images were displayed using the Slicer tool in IMOD, which allowed for 3-D rotation. When the Gag structure was optimally oriented, the image was transformed to Fourier space using an FFT algorithm within IMOD.

### On-line Availability of Tomographic Datasets

Selected tomographic datasets are available at http://www.br.caltech.edu/bjorker/ladinsky, on the Electron Microscopy Databank (http://www.emdatabank.org) under submission number 28207, or will be provided upon request.

## Supporting Information

Figure S1 **Distribution of HIV-1 virions in intestinal mucosa and lamina propria.** (A) Top: An infected cell within the general mucosa (red oval) adjacent to a venule (BV) (identified by the presence of a red blood cell; rbc). Bottom: Tomographic slice of the infected cell, displaying budding profiles and free virions (red arrowheads). N = nucleus; M = mitochondrion. (B) Top: An infected cell within the lamina propria of an intestinal villus (red oval). Bottom: Detail of the infected cell, likely a T cell because of its large nucleus. Two budding profiles (red arrowheads) were present on either side of the cell.
(TIF)

Figure S2 **Gallery of budding and free HIV-1 virions, imaged by tomography in both positive and negative stain.** Virions could be identified at all stages of egress by both staining methods. Budding virions were continuous with the plasma membrane of a host cell and confirmed as HIV-1 by the presence of a partially formed (“C-shaped”) core structure in positively-stained samples and by multi-layered Gag lattice in negatively-stained samples. Immature free virions retained these core characteristics. Mature virions were identified in both positive- and negative-stained samples by their cone-shaped or cylindrical cores.
(TIF)

Figure S3 **ImmunoEM of human antigens in uninfected and HIV-1–infected cells in BLT GALT.** (A) An uninfected T cell showing human CD4 localized to the plasma membrane (arrowheads). (B) An HIV-1–infected T cell showing CD4 localized to the endoplasmic reticulum (ER). (C) An HIV-1–infected cell, double-labeled for CD4 and HIV-1 Nef (arrowheads). Both markers localized to the ER. (D) An HIV-1–infected cell, double-labeled for CD4 and HLA class I. CD4 localized to the ER while HLA (arrow and inset) sparsely labeled the plasma membrane adjacent to an intercellular space (PM). (E) Intercellular pool of HIV-1 particles showing LFA-1 (arrows) localized to virion surfaces. (F) Overview of two cells in a region of HIV-1 infected crypt. An HIV-1 budding profile (bud) emanated from the lower cell, indicating it was actively infected. Left inset: Tomographic slice of the budding profile showing contact with the upper cell, suggesting a potential virological synapse. ICAM-1 was localized to domains of the lower cell's plasma membrane (right inset). A pool of mature HIV-1 particles (far left) may have originated from the lower infected cell (IS, intercellular space; N, nucleus).
(TIF)

Figure S4 **Negatively-stained immature virions in GALT and comparison with cryoET.** (A) Tomographic slices at the surfaces of immature virions and associated Fourier transforms. Twelve nascent virions were selected from negative-stain tomograms and viewed in slices that optimally displayed the hexagonal layer of the Gag lattice. Each slice was converted to Fourier space to confirm the hexagonal symmetry of the lattice structure. Display of the Gag lattice in both real and Fourier space demonstrated that negative-stain tomography was sufficient for resolving fine structural details of HIV-1 particles in tissue. (B,C) Comparison of negatively-stained images of HIV-1 virions in infected tissue (this study) versus cryoET of isolated virions [24]. Tomographic slices showing the hexagonal Gag lattice (left panels in B and C) and Gag layers (right panels in B and C) from immature virions in negatively-stained infected tissue (B) or in purified frozen hydrated samples (C). The width of the Gag layer in budding virions from negatively-stained infected tissues (e.g., green bar in panel B) was measured in five places in each virion, and the measurements were averaged. A green bar placed in the analogous position in panel C demonstrates the similar width of the Gag layers in immature virions in purified frozen hydrated samples. Panel C was modified from Figure 1B,D in [24] and were used with permission from Nature Publishing Group. Black bars in panel C indicate the boundaries of one ordered region of the Gag lattice; arrows point into the ordered region; the arrowheads point to regions of the membrane-MA layer that appeared bilaminar (black) or unilaminar (white).
(TIF)

Figure S5 **Intercellular pools of HIV-1.** (A) Classifications of free virion pools. Tomographic slices from a negatively-stained sample showing a pool containing 6 mature and 28 immature virions (upper panel) and a pool containing 49 mature virions with no immature particles (lower panel). Color-coded maps are shown to the right of each slice (Blue, mature virions; pink, immature virions). The immature virion indicated by a green star in the upper left panel and encircled in green in the corresponding map was associated with a “tail-like” structure that could suggest it is attached to a host cell. Careful scrutiny of this region in all three dimensions confirmed that it was indeed a free particle. (B) An isolated productively-infected cell in the smooth muscle layer of the colon near a venule (BV). Tomographic reconstructions of the cell and adjacent volumes indicated that the majority of free virions in close proximity to the cell were immature (62%), while most of the free virions in two groups distal from the cell (0.8 µm and 1.3 µm) were mature (73% and 75%, respectively). These results suggested that a given infected cell produced virions in semi-synchronous waves and that virions matured quickly once released from the host cell.
(TIF)

Figure S6 **Examples of potential cell-to-cell HIV-1 transmission in GALT.** (A) Detail of a HIV-1 budding profile in jejunum, attached to the infected cell (top) via a thin ∼50 nm long neck. The surface of the bud contacted the plasma membrane of an adjacent cell. Density at the point of association (red arrow) suggested a receptor-mediated event. (B) Overview of an actively infected region in a crypt. A pool of mature virions was present in the intercellular space (IS) between the three cells in the image. The upper (infected) cell was producing a virion bud that extended into a domain of the lower cell that was invaginating (red arrowhead). (C) Four sequential tomographic slices detailing the budding event (red arrowheads) at different levels of the tomogram.
(TIF)

Figure S7 **Galleries of HIV-1 budding profiles.** (A) Bundles of actin filaments (arrowheads) near an HIV-1 bud. (B) Examples of HIV-1 buds with a limited neck or no neck. Buds were observed at obvious points along the plasma membrane, but were often seen budding into highly convoluted surface domains that appeared to be intracellular compartments in particular views. (C) Examples of HIV-1 buds with long necks projecting from the surfaces of infected cells into the intercellular space or mucosa. Neck diameters decreased as buds approached scission. (D) Comparison of Gag lattice width in budding profiles with or without necks. Examples of tomographic slices of negatively-stained images of a budding profile with (left) or without (right) a neck. Thirty examples of each category were selected and the width of the Gag lattices within each bud was measured in five places (green bars), yielding the tabulated results. (E) Histogram of the measured budding profiles. The Gag shells of necked profiles had an average diameter of 14.7±0.9 nm while buds without necks had an average diameter of 14.6±1.1 nm.
(TIF)

Figure S8 **Galleries of budding virions displaying presumptive ESCRT structures.** (A) Gallery of six “early” budding HIV-1 virions displaying structures suggestive of ESCRT proteins. Budding profiles with relatively wide necks (>1/2 the bud diameter; likely at early stages of bud formation) were selected from negatively-stained tomograms and optimally oriented in 3-D. Each bud displayed 4–6 fine lines (red arrowheads) radiating in a spoke-like pattern from a central point in the neck, just below the forming bud. These spokes were interpreted as components of ESCRT-1 or -II, or the ESCRT adaptor ALIX, which function at early stages of neck contraction prior to scission of the nascent virion. (B,C) Gallery of “late” budding HIV-1 virions displaying structures interpreted as components of ESCRT-III. Budding profiles with neck diameters ∼1/2 that of the virion bud itself (panel B from tomograms of positively-stained, plastic-embedded GALT tissue; panel C from tomograms of negatively-stained cryosections) were selected and optimally oriented in 3-D. In each case, one to three thin lines (red arrowheads) bisected the neck region just below the forming bud. These lines were interpreted as polymerized ESCRT-III complex that formed a coil around the bud neck to facilitate scission at late stages of HIV-1 egress.
(TIF)

Movie S1 **Large-area tomographic reconstruction of an intercellular space near the edge of an HIV-1–infected crypt from BLT mouse colon.** The movie begins with a summary of the region defined in the volume: an intercellular space separating two adjacent cells (dark green and brown) near the edge of an HIV-1–infected crypt. HIV-1 buds (blue with magenta cores) were present on both cells, demonstrating that both were infected human cells. The tomographic reconstruction of the same area shown after the summary consisted of four serial 300 nm sections, each imaged as two-frame dual-axis tomograms to encompass a volume of 1.4 µm×2.9 µm×1.2 µm. The intercellular space contained ≥300 free, mature HIV-1 virions and no free immature virions. No structural evidence of direct cell-to-cell viral transmission was present in the volume. This reconstruction supports the assumption that virion pools were not necessarily a consequence of the juxtaposition of human and murine cells within the BLT mouse system. Instead pool formation and infection by free virus may occur even when adjacent cells were HIV-1 targets, further suggesting that maturation of HIV-1 occurs quickly following scission from the host cell.
(MOV)

Movie S2 **Intercalating infected cell with budding profile contacting an adjacent cell.** A large-area tomographic reconstruction comprising a 5.4 µm×3.6 µm×0.35 µm volume of a colon crypt. The region contained three cells: two crypt cells and a third cell intercalating between them. The intercalating cell is presumably a dendritic cell or macrophage due to its convoluted projections (see Figure 5B). It was HIV-1–infected and displayed four budding profiles within the reconstructed volume. One such bud (black arrow), shown in detain in the latter half of the movie, crossed the intercellular space to form a presumptive virological synapse by directly contacting the adjacent cell (red arrowheads).
(MOV)

Movie S3 **Potential ESCRT components at an early stage of HIV-1 budding.** Detail from a negatively-stained tomographic reconstruction of BLT mouse colon, showing a nascent HIV-1 virion at an early stage of budding. Slices from the tomogram move laterally through the neck region of the budding profile and reveal five fine lines (red arrowheads) radiating in a spoke-like manner from a central point in the neck. The lines can be followed through approximately half of the shown volume. These spokes are interpreted as components of host-encoded ESCRT-1 or -II, or the ESCRT adaptor ALIX. Detail from this movie is shown in Figure S8A, panel 5. Bar = 50 nm.
(MOV)

Movie S4 **Potential ESCRT components at a late stage of HIV-1 budding.** Detail from a negatively-stained tomogram of BLT mouse colon, showing an HIV-1 virion at a later stage of budding, nearer to scission. Slices from the tomogram reveal three lines that bisect the neck of the budding profile. The structures lie near the inner surface of the neck and can be followed through the last half of the shown volume. These lines are interpreted as components of ESCRT-III. Detail from this movie is shown in Figure S8C, panel 5. Bar = 50 nm.
(MOV)

Movie S5 **Comparison of a presumptive VPS4 spot and a ribosome at high magnification.** Tomographic slices through a presumptive VPS4 structure (part 1) shows a larger, pleomorphic electron-dense structure. It is distinguishable from a cytoplasmic ribosome, selected at random from the same reconstruction (part 2), which appears smaller, less dense and shows a characteristic “groove” between subunits. Examples of these structures are detailed in Figure 7. Bar = 20 nm.
(MOV)

Text S1 **Supplementary methods and extended experimental procedures.** Including immunofluorescence microscopy procedure, detailed description of EM sample preparation for both positively- and negatively-stained tissues and protocol for immuno-electron microscopy.
(DOCX)
