# Supplementary figures and images for: Electron Tomography of HIV-1 Infection in Gut-Associated Lymphoid Tissue
Source: PLoS Pathog. 2014 Jan 30;10(1):e1003899. doi: 10.1371/journal.ppat.1003899 (PMC3907528; doi:10.1371/journal.ppat.1003899)

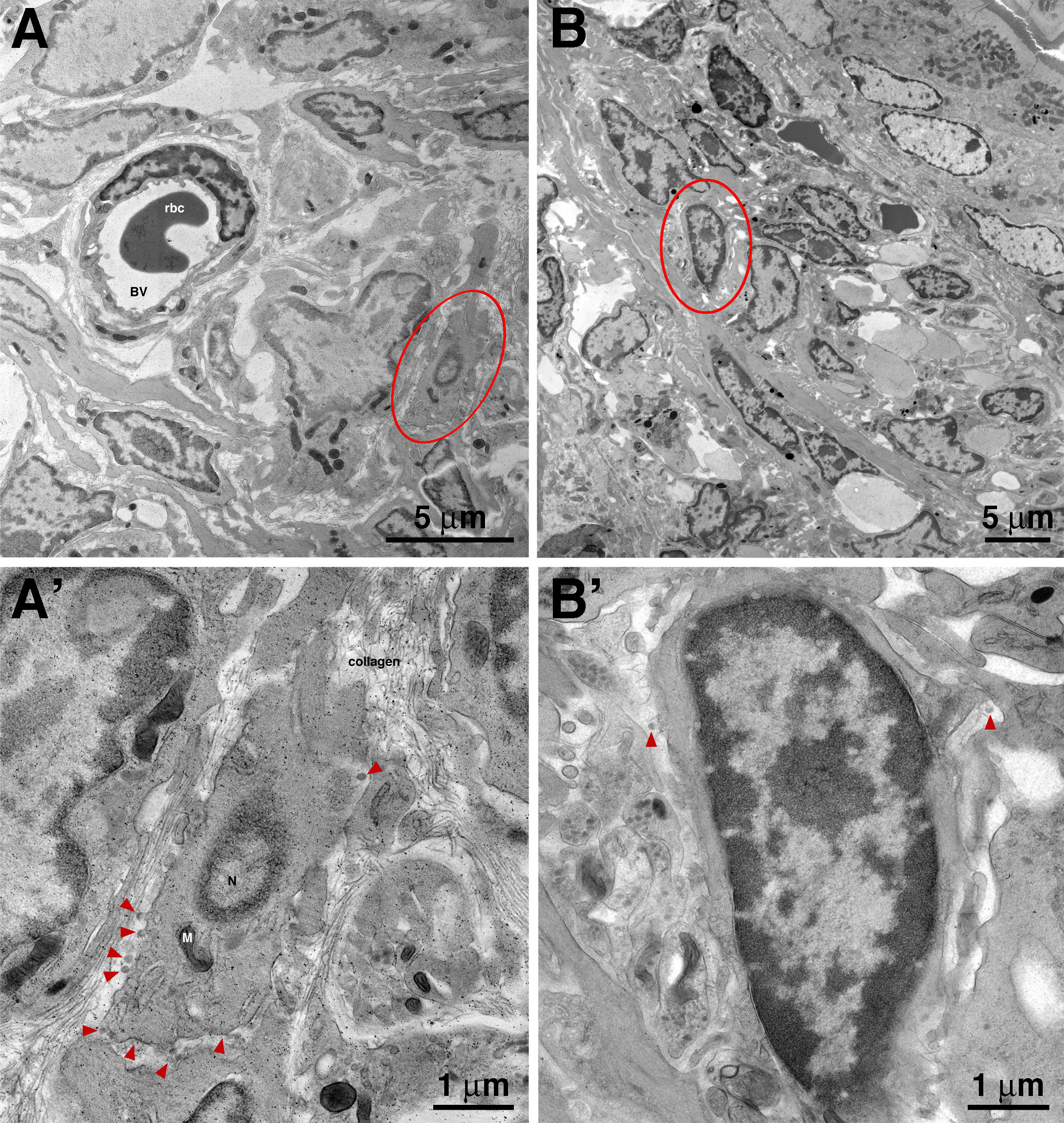

Supplement: Figure S1 — Distribution of HIV-1 virions in intestinal mucosa and lamina propria. (A) Top: An infected cell within the general mucosa (red oval) adjacent to a venule (BV) (identified by the presence of a red blood cell; rbc). Bottom: Tomographic slice of the infected cell, displaying budding profiles and free virions (red arrowheads). N = nucleus; M = mitochondrion. (B) Top: An infected cell within the lamina propria of an intestinal villus (red oval). Bottom: Detail of the infected cell, likely a T cell because of its large nucleus. Two budding profiles (red arrowheads) were present on either side of the cell. (TIF) [file ppat.1003899.s001.tif]

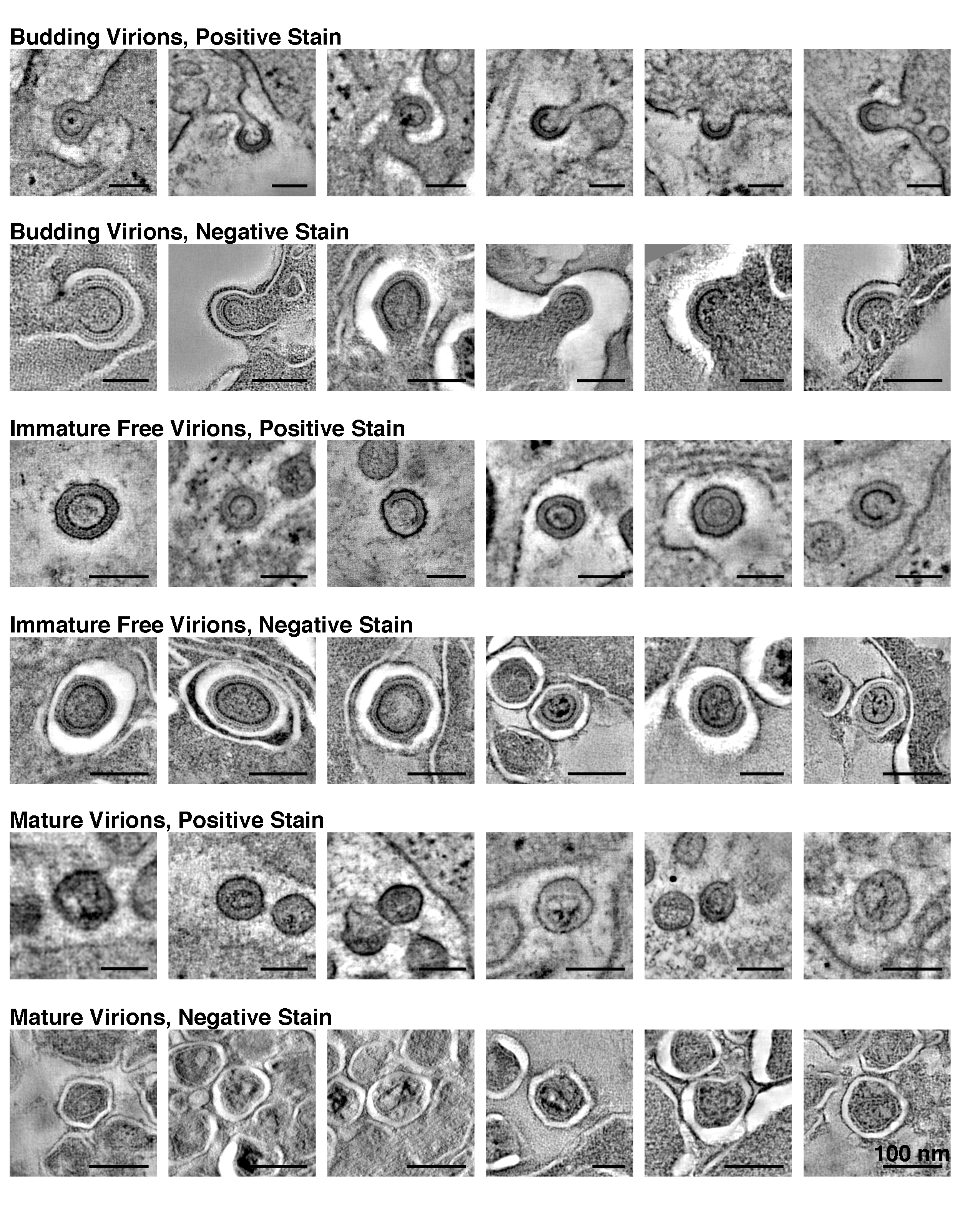

Supplement: Figure S2 — Gallery of budding and free HIV-1 virions, imaged by tomography in both positive and negative stain. Virions could be identified at all stages of egress by both staining methods. Budding virions were continuous with the plasma membrane of a host cell and confirmed as HIV-1 by the presence of a partially formed (“C-shaped”) core structure in positively-stained samples and by multi-layered Gag lattice in negatively-stained samples. Immature free virions retained these core characteristics. Mature virions were identified in both positive- and negative-stained samples by their cone-shaped or cylindrical cores. (TIF) [file ppat.1003899.s002.tif]

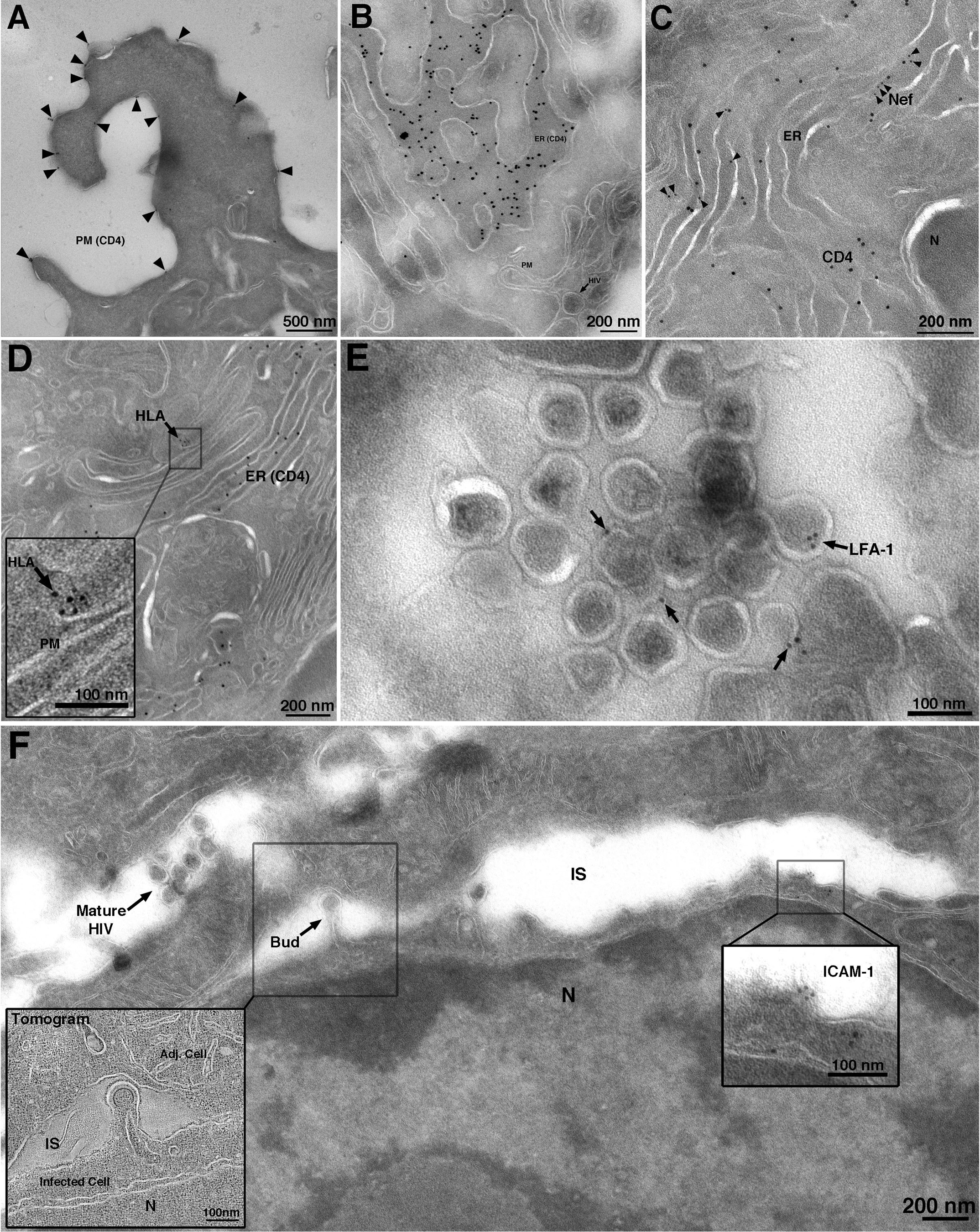

Supplement: Figure S3 — ImmunoEM of human antigens in uninfected and HIV-1–infected cells in BLT GALT. (A) An uninfected T cell showing human CD4 localized to the plasma membrane (arrowheads). (B) An HIV-1–infected T cell showing CD4 localized to the endoplasmic reticulum (ER). (C) An HIV-1–infected cell, double-labeled for CD4 and HIV-1 Nef (arrowheads). Both markers localized to the ER. (D) An HIV-1–infected cell, double-labeled for CD4 and HLA class I. CD4 localized to the ER while HLA (arrow and inset) sparsely labeled the plasma membrane adjacent to an intercellular space (PM). (E) Intercellular pool of HIV-1 particles showing LFA-1 (arrows) localized to virion surfaces. (F) Overview of two cells in a region of HIV-1 infected crypt. An HIV-1 budding profile (bud) emanated from the lower cell, indicating it was actively infected. Left inset: Tomographic slice of the budding profile showing contact with the upper cell, suggesting a potential virological synapse. ICAM-1 was localized to domains of the lower cell's plasma membrane (right inset). A pool of mature HIV-1 particles (far left) may have originated from the lower infected cell (IS, intercellular space; N, nucleus). (TIF) [file ppat.1003899.s003.tif]

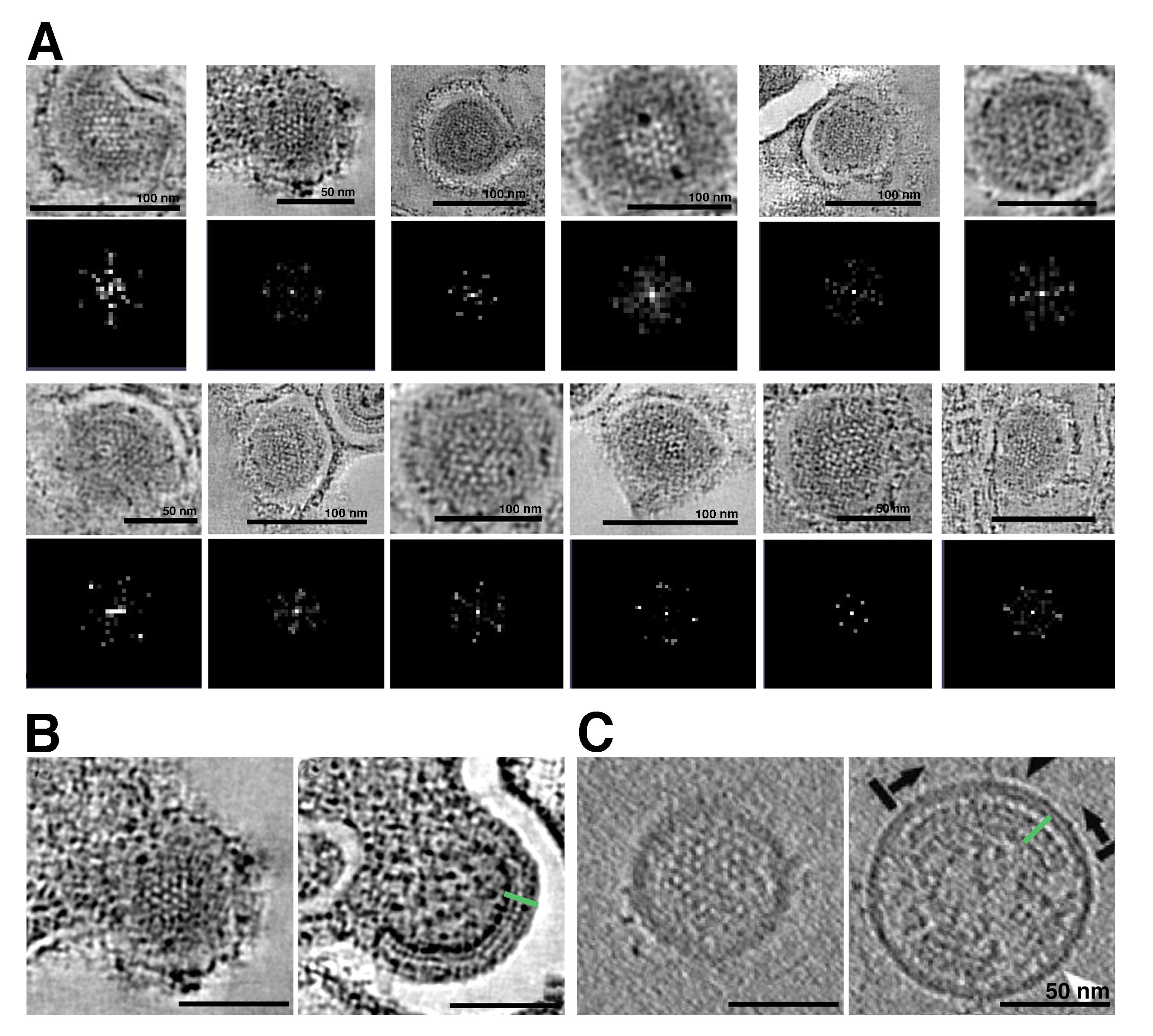

Supplement: Figure S4 — Negatively-stained immature virions in GALT and comparison with cryoET. (A) Tomographic slices at the surfaces of immature virions and associated Fourier transforms. Twelve nascent virions were selected from negative-stain tomograms and viewed in slices that optimally displayed the hexagonal layer of the Gag lattice. Each slice was converted to Fourier space to confirm the hexagonal symmetry of the lattice structure. Display of the Gag lattice in both real and Fourier space demonstrated that negative-stain tomography was sufficient for resolving fine structural details of HIV-1 particles in tissue. (B,C) Comparison of negatively-stained images of HIV-1 virions in infected tissue (this study) versus cryoET of isolated virions [24]. Tomographic slices showing the hexagonal Gag lattice (left panels in B and C) and Gag layers (right panels in B and C) from immature virions in negatively-stained infected tissue (B) or in purified frozen hydrated samples (C). The width of the Gag layer in budding virions from negatively-stained infected tissues (e.g., green bar in panel B) was measured in five places in each virion, and the measurements were averaged. A green bar placed in the analogous position in panel C demonstrates the similar width of the Gag layers in immature virions in purified frozen hydrated samples. Panel C was modified from Figure 1B,D in [24] and were used with permission from Nature Publishing Group. Black bars in panel C indicate the boundaries of one ordered region of the Gag lattice; arrows point into the ordered region; the arrowheads point to regions of the membrane-MA layer that appeared bilaminar (black) or unilaminar (white). (TIF) [file ppat.1003899.s004.tif]

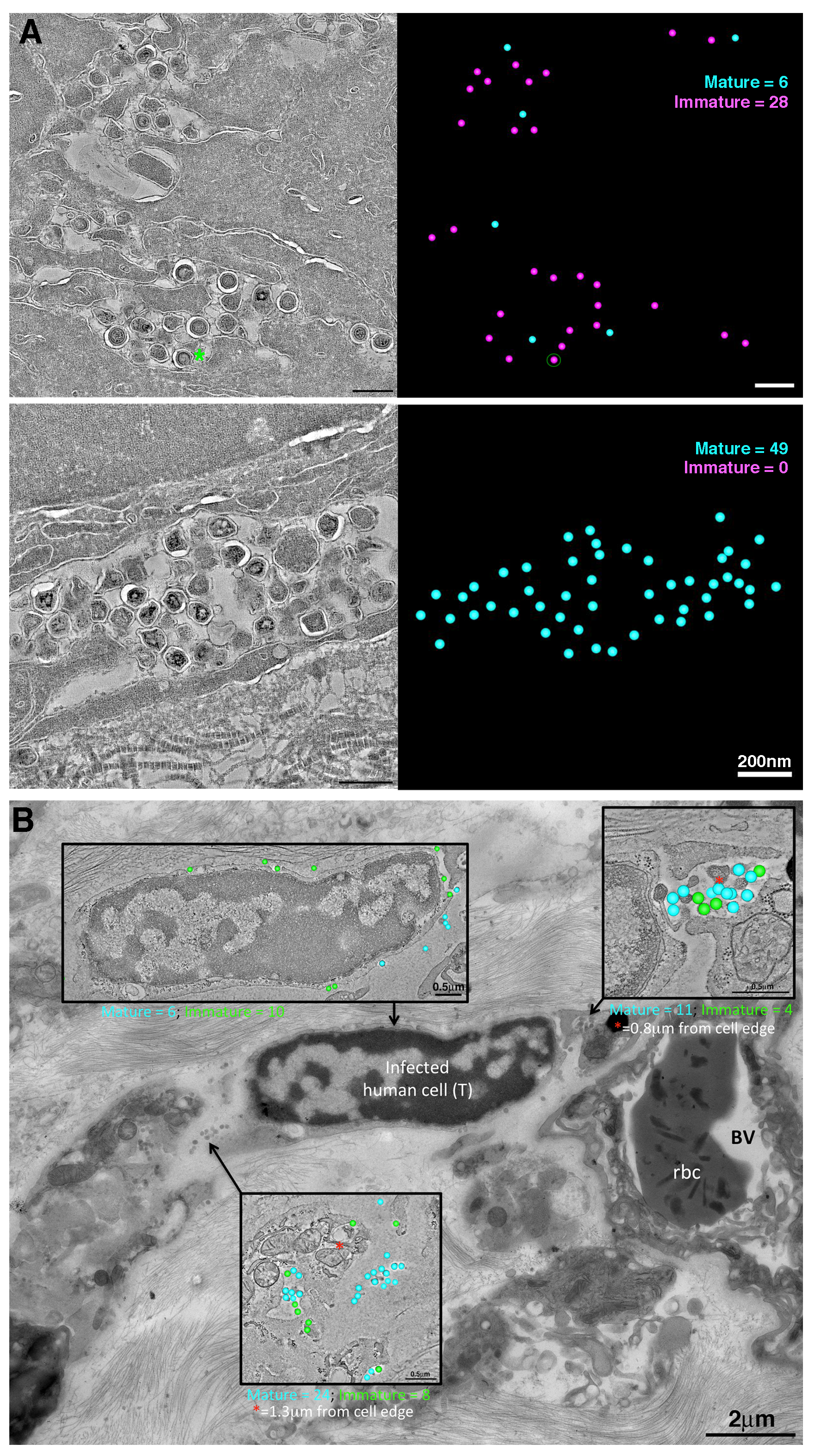

Supplement: Figure S5 — Intercellular pools of HIV-1. (A) Classifications of free virion pools. Tomographic slices from a negatively-stained sample showing a pool containing 6 mature and 28 immature virions (upper panel) and a pool containing 49 mature virions with no immature particles (lower panel). Color-coded maps are shown to the right of each slice (Blue, mature virions; pink, immature virions). The immature virion indicated by a green star in the upper left panel and encircled in green in the corresponding map was associated with a “tail-like” structure that could suggest it is attached to a host cell. Careful scrutiny of this region in all three dimensions confirmed that it was indeed a free particle. (B) An isolated productively-infected cell in the smooth muscle layer of the colon near a venule (BV). Tomographic reconstructions of the cell and adjacent volumes indicated that the majority of free virions in close proximity to the cell were immature (62%), while most of the free virions in two groups distal from the cell (0.8 µm and 1.3 µm) were mature (73% and 75%, respectively). These results suggested that a given infected cell produced virions in semi-synchronous waves and that virions matured quickly once released from the host cell. (TIF) [file ppat.1003899.s005.tif]

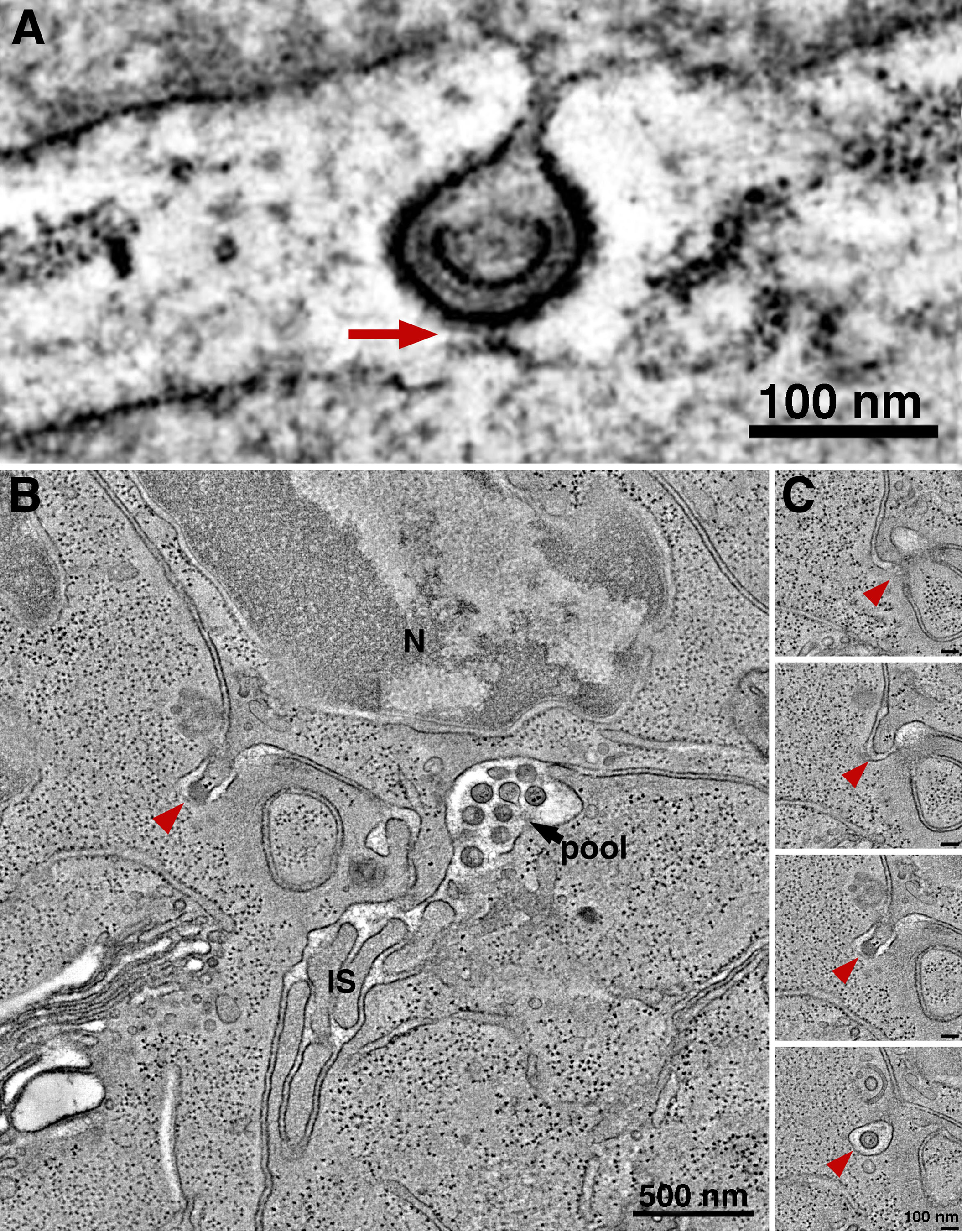

Supplement: Figure S6 — Examples of potential cell-to-cell HIV-1 transmission in GALT. (A) Detail of a HIV-1 budding profile in jejunum, attached to the infected cell (top) via a thin ∼50 nm long neck. The surface of the bud contacted the plasma membrane of an adjacent cell. Density at the point of association (red arrow) suggested a receptor-mediated event. (B) Overview of an actively infected region in a crypt. A pool of mature virions was present in the intercellular space (IS) between the three cells in the image. The upper (infected) cell was producing a virion bud that extended into a domain of the lower cell that was invaginating (red arrowhead). (C) Four sequential tomographic slices detailing the budding event (red arrowheads) at different levels of the tomogram. (TIF) [file ppat.1003899.s006.tif]

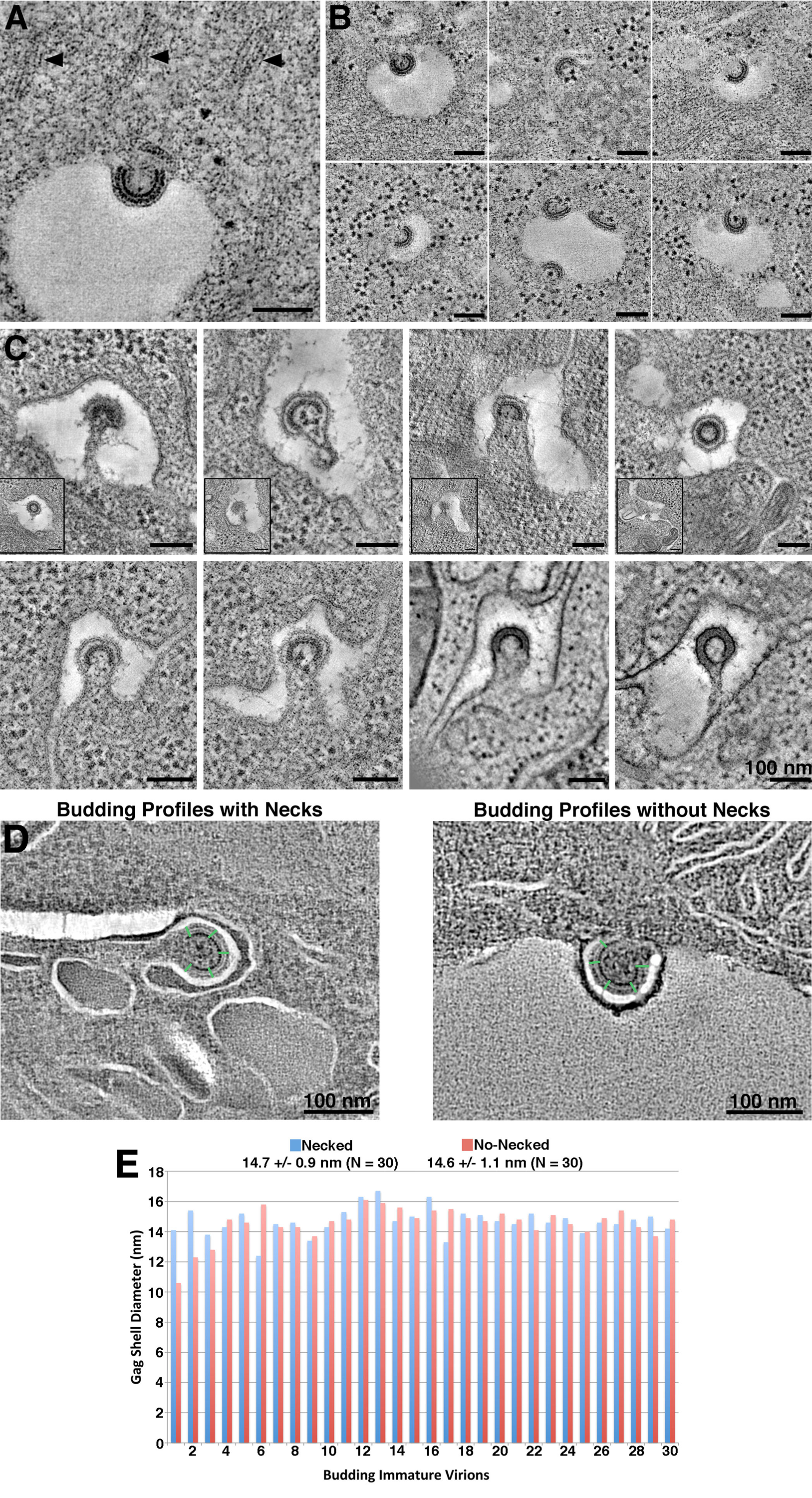

Supplement: Figure S7 — Galleries of HIV-1 budding profiles. (A) Bundles of actin filaments (arrowheads) near an HIV-1 bud. (B) Examples of HIV-1 buds with a limited neck or no neck. Buds were observed at obvious points along the plasma membrane, but were often seen budding into highly convoluted surface domains that appeared to be intracellular compartments in particular views. (C) Examples of HIV-1 buds with long necks projecting from the surfaces of infected cells into the intercellular space or mucosa. Neck diameters decreased as buds approached scission. (D) Comparison of Gag lattice width in budding profiles with or without necks. Examples of tomographic slices of negatively-stained images of a budding profile with (left) or without (right) a neck. Thirty examples of each category were selected and the width of the Gag lattices within each bud was measured in five places (green bars), yielding the tabulated results. (E) Histogram of the measured budding profiles. The Gag shells of necked profiles had an average diameter of 14.7±0.9 nm while buds without necks had an average diameter of 14.6±1.1 nm. (TIF) [file ppat.1003899.s007.tif]

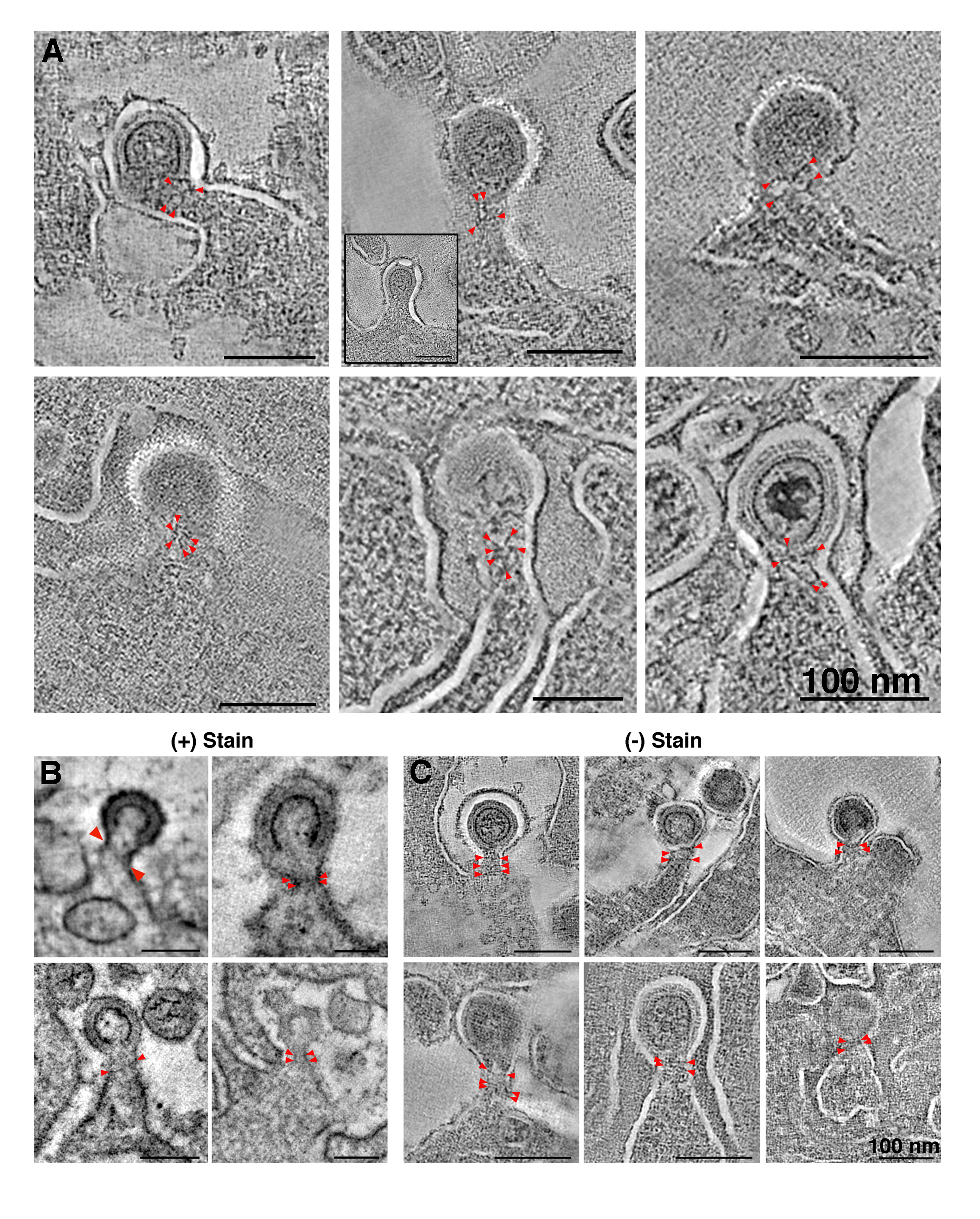

Supplement: Figure S8 — Galleries of budding virions displaying presumptive ESCRT structures. (A) Gallery of six “early” budding HIV-1 virions displaying structures suggestive of ESCRT proteins. Budding profiles with relatively wide necks (>1/2 the bud diameter; likely at early stages of bud formation) were selected from negatively-stained tomograms and optimally oriented in 3-D. Each bud displayed 4–6 fine lines (red arrowheads) radiating in a spoke-like pattern from a central point in the neck, just below the forming bud. These spokes were interpreted as components of ESCRT-1 or -II, or the ESCRT adaptor ALIX, which function at early stages of neck contraction prior to scission of the nascent virion. (B,C) Gallery of “late” budding HIV-1 virions displaying structures interpreted as components of ESCRT-III. Budding profiles with neck diameters ∼1/2 that of the virion bud itself (panel B from tomograms of positively-stained, plastic-embedded GALT tissue; panel C from tomograms of negatively-stained cryosections) were selected and optimally oriented in 3-D. In each case, one to three thin lines (red arrowheads) bisected the neck region just below the forming bud. These lines were interpreted as polymerized ESCRT-III complex that formed a coil around the bud neck to facilitate scission at late stages of HIV-1 egress. (TIF) [file ppat.1003899.s008.tif]
