# Supplementary material for: Electron Tomography of HIV-1 Infection in Gut-Associated Lymphoid Tissue
Source: PLoS Pathog. 2014 Jan 30;10(1):e1003899. doi: 10.1371/journal.ppat.1003899 (PMC3907528; doi:10.1371/journal.ppat.1003899)
Supplement: Text S1 — Supplementary methods and extended experimental procedures. Including immunofluorescence microscopy procedure, detailed description of EM sample preparation for both positively- and negatively-stained tissues and protocol for immuno-electron microscopy. (DOCX) [file ppat.1003899.s014.docx]

Supplementary Methods for

Electron tomography of HIV-1 infection in gut-associated lymphoid tissue

Mark S. Ladinsky*, Collin Kieffer*, Gregory Olson#, Douglas S. Kwon# and

Pamela J. Bjorkman*†

#Ragon Institute of MGH, MIT and Harvard

400 Technology Square

Cambridge, MA 02139

*Division of Biology and Biological Engineering 114-96

and †Howard Hughes Medical Institute

California Institute of Technology

1200 East California Boulevard

Pasadena, CA 91125

Supplementary Methods

**Extended Experimental Procedures**

**Immunofluorescence (IF)**

The following antibodies were used to localize human or HIV antigens: rabbit polyclonal anti-CD3 (A0452; Dako North America, Inc., Carpinteria, CA), mouse monoclonal anti-CD4 (VP-C319; Vector Laboratories, Inc., Burlingame, CA), mouse monoclonal CD68 (M0876; Dako), mouse monoclonal CD209/DC-SIGN (ab59192; Abcam Inc., Cambridge, MA) and mouse monoclonal HIV-1 anti-p24 (M0857; Dako). Fluorescently-conjugated secondary antibodies included AlexaFluor-568 goat anti-rabbit (A11011; Invitrogen, Grand Island NY) and goat anti-mouse (A21144; Invitrogen).

Formalin fixed, paraffin embedded tissue from colon and small bowel samples of HIV-1–infected and uninfected mice were cut in 4 µm sections. Antigen retrieval was carried out in a Decloaking Chamber (BioCare Medical), either in EDTA pH 8 (Invitrogen) for CD3, CD4, and DC-SIGN, or in citrate buffer pH 6 (Invitrogen) for CD68 and p24. All CD antigen antibodies were incubated 1 hr at room temperature. Anti-p24 was incubated overnight at 4ºC. After primary incubation, the slides were washed five times in Tris buffered saline with 0.05% Tween-20 (TBST). Secondary antibodies were applied for 30 minutes at room temperature. After another five washes with TBST, Tyramide signal amplification (TSA) reagent (Perkin Elmer) (diluted 1:50 in TSA diluent) was added to the slides with horseradish peroxidase (HRP) for 5 min at room temperature. The slides were washed again five times with TBST, and then ProLong Gold Antifade Reagent with Dapi (Invitrogen) was added just before cover slipping. Slides were imaged with a Zeiss Axio A1 microscope and a Zeiss MIRAX MIDI slide scanner.

**ET sample preparation**

For positively-stained samples, tissue segments were rinsed in ice-cold cacodylate buffer (0.1M sodium cacodylate trihydrate + 5% sucrose) immediately after sacrifice, and then fixed with ice-cold 3% glutaraldehyde, 1% paraformaldehyde (Electron Microscopy Sciences, Port Washington PA). Following ≥1 hr in fixative, tissue segments (now non-infectious) were rinsed twice with cacodylate buffer, and then trimmed to ~1 mm^3^ blocks and placed into aluminum or brass freezing planchettes (Ted Pella, Inc.) that were prefilled with cacodylate buffer containing 10% Ficoll. Samples were rapidly frozen in a high-pressure freezer (HPM-010, Bal-Tec) and transferred to liquid nitrogen. The planchettes were opened under liquid nitrogen and placed in cryo tubes containing 2 ml of 2.5% OsO_4_, 0.05% uranyl acetate in acetone, and then transferred to an AFS freeze-substitution machine (Leica Microsystems, Austria). Samples were freeze-substituted for 60 hours at -90°C, and then warmed to -20°C over 10 hours. Following 24 hr at -20°C, the samples were warmed to room temperature, rinsed with acetone and infiltrated with Epon-Araldite resin (Electron Microscopy Sciences). Tissue pieces were flat-embedded between two Teflon-coated glass slides and polymerized at 60°C for 48 hr.

Suitable blocks of flat-embedded samples were excised with a scalpel, oriented and remounted onto a plastic sectioning stub using a two-part epoxy adhesive (907, Miller-Stephenson Chemical Co., Danbury, CN). 200 or 300 nm serial sections were cut with a UC6 ultramicrotome (Leica Microsystems) using a diamond knife with a 45° included angle (Diatome, Ltd., Switzerland), transferred to Formvar-coated copper-rhodium 1 mm slot grids (Electron Microscopy Sciences) and stained with 3% uranyl acetate and lead citrate. 10 or 15 nm colloidal gold particle fiducials were placed on both grid surfaces, and the grids were stabilized with evaporated carbon.

For negatively-stained samples, issues were fixed with ice-cold 10% paraformaldehyde, 5% sucrose, 0.1M HEPES in PBS for 12–24 hours, after which they were cut into blocks of <1 mm^3^ and infiltrated with 2.1 M sucrose in PBS over 10 hr (solution changes ever hr). Each tissue block was then affixed to an aluminum sectioning stub, drained of excess solute and quickly frozen in liquid nitrogen. Cryosections (90 nm) were cut at -110°C with a UC6/FC6 cryoultramicrotome using a Cryo-Immuno diamond knife with a 35° included angle (Diatome Ltd., Switzerland). Cryosections were transferred in a wire loop containing a drop of 2.3 M sucrose in PBS to Formvar-coated, carbon-coated, glow-discharged 100-mesh copper/rhodium grids, warmed to room temperature, and then negatively stained and embedded with 1% uranyl acetate, 1% methylcellulose in dH_2_O for 20 min. The stained grids were air-dried in wire loops overnight.

**ImmunoEM**

Samples for immunoEM were prepared as described for negatively-stained samples, but were labeled with antibodies prior to the negative staining step. Rabbit polyclonal anti-CD4 (ab79040), mouse monoclonal anti-HIV-1 Nef (ab42356), mouse monoclonal anti-HLA class I (ab23755) and mouse monoclonal anti-HIV-1 p24 (ab9072) were purchased from Abcam Inc., Cambridge, MA. Rabbit polyclonal antibodies against CHMP1b, CHMP2A and ALIX were gifts from Wesley I. Sundquist, University of Utah. 2G12, a human monoclonal antibody against HIV-1 gp120, was expressed in HEK293 cells, and the monomeric fraction was purified as described ([West et al., 2009](#_ENREF_3)). Colloidal gold (10 and 15 nm) conjugated anti-mouse, anti-rabbit and anti-human secondary antibodies were purchased from Ted Pella, Inc. (Redding, CA). Nonspecific antibody binding sites were blocked by incubating sections in 10% fetal calf serum in PBS for 30 min. Sections were labeled with primary antibodies diluted in PBS + 5% calf serum for 2 hours, rinsed 4x with PBS, then incubated with 10 nm or 15 nm colloidal gold-conjugated secondary antibodies (Tell Pella, Inc.) for an additional 2 hours. Sections were rinsed 4x with PBS, 3x with dH_2_O, and then negatively stained and embedded as described above.

**References**

Kremer, J.R., Mastronarde, D.N., and McIntosh, J.R. (1996). Computer visualization of three-dimensional image data using IMOD. J Struct Biol *116*, 71-76.

Mastronarde, D.N. (2008). Correction for non-perpendicularity of beam and tilt axis in tomographic reconstructions with the IMOD package. J Microsc *230*, 212-217.

West, A.P., Jr., Galimidi, R.P., Foglesong, C.P., Gnanapragasam, P.N., Huey-Tubman, K.E., et al. (2009). Design and expression of a dimeric form of human immunodeficiency virus type 1 antibody 2G12 with increased neutralization potency. J Virol *83*, 98-104.

Wright, E.R., Schooler, J.B., Ding, H.J., Kieffer, C., Fillmore, C., et al. (2007). Electron cryotomography of immature HIV-1 virions reveals the structure of the CA and SP1 Gag shells. EMBO J *26*, 2218-2226.

Zhu, P., Liu, J., Bess, J., Jr., Chertova, E., Lifson, J.D., et al. (2006). Distribution and three-dimensional structure of AIDS virus envelope spikes. Nature *441*, 847-852.
